# Risk prediction models for cardiac rupture after acute myocardial infarction: a systematic review and meta-analysis

**DOI:** 10.3389/fcvm.2026.1721103

**Published:** 2026-02-11

**Authors:** Yijun Mao, Qiang Liu, Hui Fan, Xueqian Ouyang, Xiaojuan Wang

**Affiliations:** 1 Department of Nursing, Xianyang Central Hospital, Xianyang, Shaanxi, China; 2 Department of Orthopedic Surgery, Xianyang Central Hospital, Xianyang, Shaanxi, China

**Keywords:** acute myocardial infarction, cardiac rupture, meta-analysis, prediction model, systematic review

## Abstract

**Background:**

Cardiac rupture (CR) is a catastrophic complication of acute myocardial infarction (AMI), accounting for 10%–20% of AMI-related deaths despite its low incidence. Several risk prediction models have been developed, but their methodological quality and clinical applicability remain uncertain. This study systematically reviewed and quantitatively synthesized existing prediction models for CR post-AMI.

**Methods:**

We searched PubMed, Embase, Web of Science, Cochrane Library, CNKI, and Wanfang databases from inception to August 2025. Studies developing or validating risk prediction models for CR after AMI were eligible. Data extraction followed the CHARMS checklist, and methodological quality was assessed with PROBAST. A meta-analysis was performed to pool model discrimination (C-statistic) and evaluate predictors of CR. Subgroup analyses explored heterogeneity by publication period, study design, population, sample size, and validation approach.

**Results:**

Ten studies (2017–2024) involving 74–11,603 patients were included. Among them, nine studies reported C-statistics (AUC) along with their confidence intervals (CIs), which were suitable for quantitative synthesis (Meta-analysis). The pooled C-statistic of CR prediction models was 0.83 (95% CI: 0.78–0.89), though with high heterogeneity (*I*^2^ = 88%). Consistently robust predictors included advanced age (OR = 2.26), female sex (OR = 2.43), higher Killip grade (OR = 3.58), elevated heart rate (OR = 2.29), lower LVEF (OR = 1.46), and absence of emergency PCI (OR = 0.37, protective). Most studies exhibited methodological flaws, including small events-per-variable ratios, univariate-based predictor selection, inadequate handling of missing data, and limited external validation.

**Conclusion:**

Existing models demonstrate promising discriminatory ability for predicting CR after AMI but are undermined by substantial methodological limitations. Age, sex, Killip grade, LVEF, and PCI status represent robust predictors that should inform future consensus-based models. Large-scale, prospective, and externally validated studies are urgently needed to develop reliable tools for clinical risk stratification and targeted prevention of this lethal complication.

**Systematic Review Registration:**

https://www.crd.york.ac.uk/PROSPERO/view/CRD420251105703, PROSPERO CRD420251105703.

## 1 Introduction

Cardiac rupture (CR) is a catastrophic complication of acute myocardial infarction (AMI), accounting for 10%–20% of AMI-related deaths despite its low absolute incidence (0.5%–3%) (1). The abrupt onset and high fatality rate (>90%) make CR a critical yet understudied clinical challenge (2–4). Although early identification of high-risk patients could guide intensive monitoring and intervention, no consensus exists on optimal risk stratification tools for CR post-AMI (5). Several risk prediction models for CR have been proposed, but these models vary widely in predictor selection, methodological quality, and generalizability (6). Many lack external validation or fail to address temporal changes in AMI management. A systematic synthesis of existing models is urgently needed to (i) evaluate their discriminatory performance and clinical applicability, (ii) identify robust predictors across heterogeneous populations, and (iii) highlight methodological gaps in model development. Such an analysis will inform the development of consensus-based models and optimize targeted prevention strategies. We therefore conducted a systematic review and meta-analysis to critically appraise and quantitatively synthesize risk prediction models for CR after AMI, following PRISMA and CHARMS guidelines.

## 2 Methods

This systematic review and meta-analysis were conducted in accordance with the Preferred Reporting Items for Systematic Reviews and Meta-Analyses (PRISMA), the Critical Appraisal and Data Extraction for Systematic Reviews of Prediction Modelling Studies (CHARMS) checklist, and the Transparent Reporting of a Multivariable Prediction Model for Individual Prognosis or Diagnosis (TRIPOD) guidelines (see Supplementary Tables S1–3) (7–10). The review protocol was registered with PROSPERO (registration number: CRD420251105703). The eligibility conditions for the reviewed investigations are defined following the PICOS approach (Table 1) (11).

**Table 1:** Selection criteria of predictive modelling studies in PICOS format.

Criteria	Participants (P)	Intervention (I)	Comparison (C)	Outcomes (O)	Timeframe (T)	Settings (S)	Other limitations
Inclusion criteria	Patients with AMI	Studies that developed or validated risk prediction models for cardiac rupture post-AMI using traditional statistical methods or ML algorithms. For quantitative synthesis, priority was given to studies that reported the C-statistic (AUC), including: (a) studies that performed formal validation, and (b) studies involving only model development but reporting the C-statistic of the development set	N/A	C-statistic, accuracy, sensitivity, specificity, Hosmer-Lemeshow test, DCA, et al.	From each database's inception to July 31, 2025	All clinical settings, including emergency departments, cardiology wards, and intensive care units, across high-income, middle-income, and low-income countries were included	Language = English and Chinese
Exclusion criteria	Non-AMI-related cardiac rupture (traumatic/iatrogenic)	Studies lacking explicit prediction model descriptions					

AMI, acute myocardial infarction; DCA, decision curve analysis; ML, machine learning; N/A, not applicable.

### 2.1 Participants (P)

Studies involving patients diagnosed with AMI who subsequently developed cardiac rupture or were at risk of cardiac rupture. No restrictions were placed on age, gender, ethnicity, or geographic location of participants.

### 2.2 Intervention (I)

Studies that developed or validated risk prediction models for cardiac rupture post-AMI using traditional statistical methods or machine learning algorithms. For the quantitative synthesis (meta-analysis) of model performance, our primary analysis focused on studies that reported discrimination metrics, specifically the C-statistic (AUC). This included two categories of studies: (a) those that conducted formal validation (internal or external), and (b) those that only involved model development but reported the C-statistic from the development set. Studies lacking explicit prediction model descriptions will be excluded.

### 2.3 Outcomes (O)

The effects and properties of prediction models, measured by discrimination, calibration, and clinical utility.

### 2.4 Timeframe (T)

The database search encompassed records from each database's inception through July 31, 2025.

### 2.5 Settings (S)

All clinical settings, including emergency departments, cardiology wards, and intensive care units, across high-income, middle-income, and low-income countries were included. Studies published in English or Chinese were eligible for inclusion.

#### 2.5.1 Search strategy

A comprehensive literature search was conducted using PubMed, Web of Science, Cochrane Library, Embase, CNKI, and Wanfang databases, covering publications from database inception through August 1, 2025. Details of the search strategy, including keywords, and the inclusion and exclusion criteria, are provided in the Supplementary Table S4.

#### 2.5.2 Data extraction

The extracted variables were aligned with the CHARMS framework and encompassed:

- Participants (e.g., proportion with cardiac rupture).
- Study design (e.g., prospective or retrospective cohort, sample size).
- Outcome measures (e.g., definition).
- Model development (e.g., method for selection of predictors, validation method).
- Model performance (e.g., c-statistic).

#### 2.5.3 Risk of bias assessment

The risk of bias (ROB) for each included study was assessed using the Prediction model Risk Of Bias ASsessment Tool (PROBAST) (12).

#### 2.5.4 Data synthesis and statistic analysis

A meta-analysis was conducted to assess the discriminative performance of cardiac rupture post infarction prediction model, as measured by the area under the receiver operating characteristic curve (AUC). To be eligible for inclusion, studies were required to report or allow calculation of the C-statistic and corresponding 95% confidence intervals (CIs) for both models and traditional risk scores. To comprehensively evaluate the model performance, we conducted three independent meta-analyses of the C-statistic (AUC), following the inclusion criteria outlined below: (a) development set performance analysis: only studies that explicitly reported the C-statistic along with its 95% CI in the development dataset were included; (b)validation set performance analysis: only studies that explicitly reported the C-statistic along with its 95% CI in an independent internal or external validation dataset were included; (c)integrated performance analysis: to maximize data utilization, a combined analysis was performed. This analysis prioritized the inclusion of validation set C-statistics; for studies that did not perform validation, the development set C-statistic was used instead. Studies that did not report the 95% CI were excluded from all quantitative pooled analyses. Heterogeneity was evaluated using the Q-test and the *I*^2^ statistic. Substantial heterogeneity was defined as *I*^2^ > 50% and Q-test *P* ≤ 0.1, warranting the use of a random-effects model. In contrast, low heterogeneity (*I*^2^ ≤ 50% and Q-test *P* > 0.1) supported the use of a fixed-effects model. For analyses with significant heterogeneity (*I*^2^ > 50%), subgroup analyses were conducted to explore potential sources of variability, stratified by publication date, types of study, participants, sample size, outcome and validation approach. Funnel plot asymmetry was assessed both visually and statistically using Egger's linear regression test, with *P* < 0.05 considered indicative of potential publication bias. All analyses were performed in Review Manager (version 5.4; The Cochrane Collaboration, London, UK) and R (version 4.4.1; R Foundation for Statistical Computing, Vienna, Austria) using the meta and metafor packages.

## 3 Results

### 3.1 Search results

**Figure 1:**
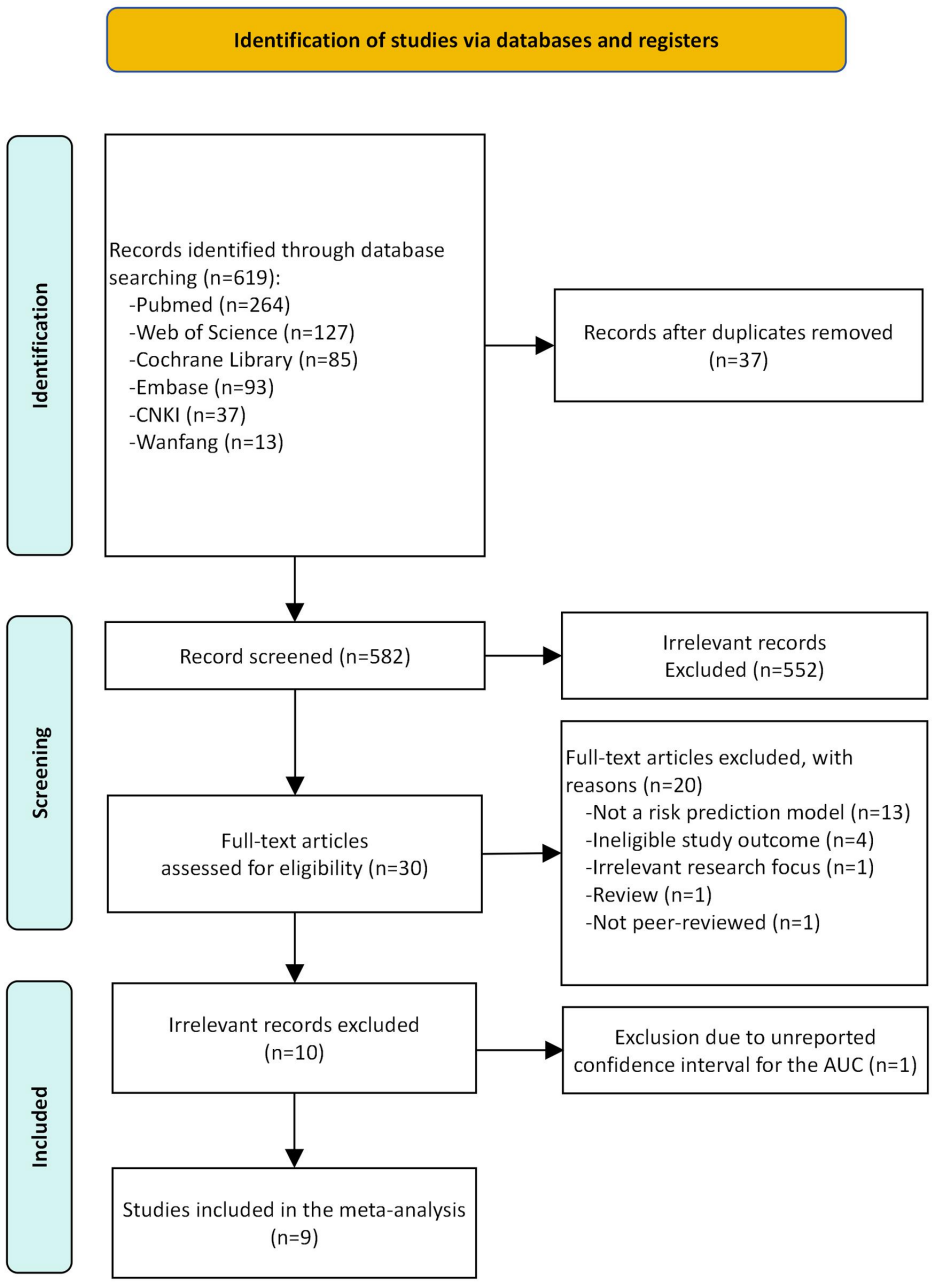
The preferred reporting items for systematic review and meta-analysis (PRISMA) flowchart of studies.

Following the removal of duplicates, the literature search yielded 582 unique records. Of these, 10 studies met the inclusion criteria and were included in the systematic review (6, 13–21). However, as one study (18) reported the AUC without its 95% CI, it was excluded from quantitative synthesis. Consequently, the meta-analysis on model discriminative ability was subsequently performed based on the remaining nine studies. Figure 1 PRISMA flow diagram illustrates the complete process from identification to inclusion of studies in the systematic review. Detailed reasons for the exclusion of full-text articles are provided in Supplementary Table S5.

### 3.2 Included studies

This review included 10 studies published between 2017 and 2024. Nine studies were retrospective in design, with one study utilizing a retrospective approach for model development and a prospective approach for validation. Sample sizes ranged from 74 to 11,603 patients. Among the included studies, 2 involved only model development, 3 conducted internal validation, 2 performed external validation, and 3 implemented both internal and external validation. The most commonly reported models were the one developed by Qian et al. (*n* = 3) and the GRACE score (*n* = 2). A detailed summary of study characteristics is provided in Table 2; Supplementary Table S6.

**Table 2:** Basic characteristics of the included studies.

Author (year)	Type	Source data	Region	Patient recruitment years	Participants	Main outcome	CR cases/sample size (%)	EPV
Abulimiti et al. (2022) (14)	D	RC	China	2013–2019	STEMI	CR	37/74^a^	1.0
Bai (2024) (15)	D/V	RC	China	2015–2023	AMI	FWR (ACC)	55/11,603 (0.47%)	2.8
Fu et al. (2019) (6)	D/V	RC	China	2010–2017	AMI	CR	53/7985 (0.6%)	1.3
Luo et al. (2022) (17)	D/V	RC	China	2013–2020	AMI	CR (ACC)	126/5490 (2.3%)	4.2
Qian et al. (2017) (13)	D/V	RC/PC^b^	China	2012–2013	STEMI	CR (ESC)	238/11,234 (2.12%)	9.2
Wu et al. (2024) (19)	D/V	RC	China	2015–2019	STEMI	CR (ACC)	91/5,412 (1.68%)	1.9
Wubuli et al. (2020) (16)	D/V	RC	China	2013–2018	STEMI	CR (ESC)	167/9,053 (1.84%)	4.6
Yan (2021) (20)	D/V	RC	China	2012–2019	STEMI	CR (ACC)	68/3,756 (1.81%)	3.2
Yisimitila et al. (2023) (18)	D	RC	China	2013–2019	STEMI	CR	38/151^a^	1.7
Zhang et al. (2024) (21)	D/V	RC	China	2010–2023	AMI	FWR (ACC)	73/233^a^	2.9

ACC, American College of Cardiology; AMI, acute myocardial infarction; CR, cardiac rupture; D, development study; EPV, events per variable; ESC, European Society of Cardiology; FWR, free wall rupture; PC, prospective study; RC, retrospective study; STEMI, ST-segment elevation myocardial infarction; V, validation study.

a The sample sizes of studies are subsets of the total eligible population; thus, the reported proportions are not indicative of the true incidence of cardiac rupture and are provided for descriptive purposes only.

b The study developed the model using a retrospective design and subsequently validated it in a prospective cohort.

### 3.3 Study characteristics

All models were based on logistic regression. Six studies did not specify their approach to handling missing data. The remaining four studies excluded patients with substantial missing data or variables with high levels of missingness to preserve data integrity.

The number of variables included per model ranged from 4 to 9, with a total of 23 unique variables identified across all studies. The most common predictor used in the predictive model was age, which nine out of ten studies included in their model, followed by female sex, where five out of ten studies used it, and Killip grade, which was included in three studies. Other predictors were heart rate, systolic blood pressure, BMI, comorbidities, clinical indicators, laboratory values, and treatment-related factors (Supplementary Table S7). Among the predictors involved in this study, the top five predictors are age, female sex, heart rate, Killip grade, and LVEF.

Validation methodologies are summarized in Table 3. Two studies did not report any validation approach. Among the remaining studies, hold-out and geographic validation were the most common method, used in 50% of studies (either alone or combined with other methods). Bootstrap validation accounted for 25% of studies. Temporal validation was used in 12.5% of studies.

**Table 3:** Domains of predictors and performance of cardiac rupture after acute myocardial infarction risk prediction models.

Author (year)	Missing data handling	Continuous variable processing method	Variable selection	Model development method	Calibration method	Validation method	Final predictors	Model performance	Model presentation
Abulimiti et al. (2022) (14)	NA	Made binary using cutoff points	Univariate logistic regression	LR	Hosmer-Lemeshow test	NI	Age Female Emergency PCI LVEF (<48%) LVESD (<50mm)	A: 0.930 (0.871, 0.988)	Risk score
Bai (2024) (15)	Direct deletion	Made binary using cutoff points	Univariate logistic regression	LR	Hosmer-Lemeshow test	Hold-out Validation	CRP (>62.9 mg/L) Gensini score (>46.5) LVEF (<48%) Comorbid pericardial effusion Post-procedural TIMI flow (<grade 3)	A: 0.907 (0.703, 0.996) B: 0.858 (0.791, 0.924)	Risk score
Fu et al. (2019) (6)	Direct deletion	Made binary using cutoff points	Step-wise backward	LR	Hosmer-Lemeshow test	No internal validation & Geographic Validation	Female No pPCI treatment LVEF(<40%) Heart rate (≥94 beats/min) BMI (<25 kg/m^2^) Age (≥ 68 years)	A: 0.843 (0.781, 0.905)^a^	Risk score
Luo et al. (2022) (17)	Direct deletion	Made binary using cutoff points	Univariate logistic regression	LR	Hosmer-Lemeshow test	No internal validation & Geographic Validation	Age (≥63 years) Female Systolic blood pressure (≤120 mmHg) Heart rate (≥100 beats/min) NPAR (≥2) Creatinine (≥106 μmol/L) Calcium concentration (≤2.2 mmol/L) Emergency PCI Oral *β*-blockers	A: 0.906 (0.865, 0.948) B: 0.785 (0.696, 0.875)	Risk score
Qian et al. (2017) (13)	NA	Made binary using cutoff points	Stepwise backward selection	LR	NA	Temporal validation & Geographic Validation	Age (≥65 years) Female Heart rate (≥80 bpm) Anterior myocardial infarction Hemoglobin (≤120 g/L) WBC (≥10 × 10^9^/L) Time-to-hospital admission (≥12 h)	B: 0.78 (0.73, 0.84)^b^	Risk score
Wu et al. (2024) (19)	NA	Continuity	LASSO	LR	Hosmer-Lemeshow test	Bootstrap	Age Killip grade First medical contact time WBC Emergency PCI ACEI/ARB within 24 h	A: 0.946 (0.927, 0.961) B: 0.947 (0.927, 0.959)	Nomogram
Wubuli et al. (2020) (16)	NA	Made binary using cutoff points	Forward-logistic regression stepwise	LR	Hosmer-Lemeshow test	Hold-out Validation & Geographic Validation	History of stroke Female Age No pPCI treatment Killip grade	A: 0.771 (0.723, 0.819) B: 0.758 (0.682, 0.835)	Risk score
Yan (2021) (20)	Direct deletion	Made binary using cutoff points	Univariate logistic regression	LR	Hosmer-Lemeshow test	Hold-out Validation & Bootstrap	Age WBC Killip grade Time-to-hospital admission (≥12 h)	A: 0.861 (0.801, 0.922) B: 0.829 (0.719, 0.939)	Nomogram
Yisimitila et al. (2023) (18)	NA	NI	Univariate logistic regression	LR	NI	NI	Age LVEF CK-MB No pPCI treatment	A: 0.971	Nomogram
Zhang et al. (2024) (21)	NA	Made binary using cutoff points	Univariate logistic regression	LR	Hosmer-Lemeshow test	Hold-out Validation	Pericardial effusion Age (≥62 years) CRP (≥23 mg/L) Neutrophil percentage (≥82%)	A: 0.924 (0.879, 0.968) B: 0.725 (0.613, 0.837)	Risk score

ACEI, angiotensin-converting enzyme inhibitor; ARB, angiotensin II receptor blocker; AUC, area under the curve; B, validation cohort; B1, internal validation cohort; B2, external validation cohort; BMI, body mass index; CK-MB, creatine kinase-MB; CRP, c-reactive protein; LR, logistic regression; LASSO, least absolute shrinkage and selection operator; LVEF, left ventricular ejection fraction; LVESD, left ventricular end-systolic diameter; NA, not assessed; NI, no information; NPAR, neutrophil percentage to albumin ratio; PCI, percutaneous coronary intervention; pPCI, primary percutaneous coronary intervention; TIMI, thrombolysis in myocardial infarction; WBC, white blood cell count.

Note on AUC interpretation: AUC = 0.5–0.7 indicates poor discrimination, 0.7–0.8 moderate discrimination, 0.8–0.9 good discrimination, and 0.9–1.0 excellent discrimination.

a This study performed internal validation using the Bootstrap method. The reported C-statistic reflects performance in the derivation cohort, and the original literature did not provide performance metrics for the validation cohort.

b This study reported only the C-statistic for the internal validation cohort. Performance data for the development set were not explicitly provided in the original publication.

### 3.4 Meta-analysis of predictors for cardiac rupture

A total of nine risk factors were identified from the included studies and were meta-analyzed. The study by Yisimitila et al. (18) was excluded from this meta-analysis due to failure to report the confidence interval for the AUC. The pooled estimates, heterogeneity statistics, and definitions of comparison for each factor are summarized in Table 4. The corresponding forest plots are available in the Supplementary Figures S1A–J.

**Table 4:** Key findings from the meta-analysis of cardiac rupture after acute myocardial infarction risk factors.

Factors	No studies	OR (95%CI)	*I*^2^ (%)	Tau^2^	Comparison	Egger's p	Subgroup (Participants)
Age	9	2.26 (1.29–3.95)	95	0.392	old vs. young	NA	AMI: OR = 4.75 (0.81–27.76) STEMI: OR = 1.77 (0.96–3.26)
Emergency PCI	6	0.37 (0.20–0.70)	29	<0.0001	yes vs. no	NA	AMI: OR = 0.39 (0.08–1.89) STEMI: OR = 0.19 (0.02–1.65)
Female gender	5	2.43 (1.92–3.07)	0	0.014	female vs. male	NA	AMI: OR = 2.75 (1.40–5.41) STEMI: OR = 2.37 (1.40–3.99)
LVEF	4	1.46 (1.28–1.67)	0	0	low vs. high	NA	AMI: OR = 1.63 (1.52–1.73) STEMI: OR = 1.42 (0.71–2.84)
Heart rate	3	2.29 (1.72–3.06)	0	0	high vs. low	NA	AMI: OR = 2.76 (0.94–8.17)
WBC	3	1.47 (0.46–4.71)	95	0.206	high vs. low	NA	STEMI: OR = 1.47 (0.46–4.71)
Time-to-hospital admission	3	1.64 (0.45–5.90)	93	0.226	long vs. short	NA	STEMI: OR = 1.64 (0.45–5.90)
Killip grade	3	3.58 (3.46–3.71)	0	0.996	high vs. low	NA	STEMI: OR = 3.58 (3.46–3.71)
CRP	2	Not pooled^a^	94	4.506	high vs. low	NA	Not pooled^a^
Pericardial effusion	2	Not pooled^a^	65	0.612	yes vs. no	NA	Not pooled^a^

AMI, acute myocardial infarction; CI, confidence interval; CRP, c-reactive protein; LVEF, left ventricular ejection fraction; NA, not applicable; OR, odds ratio; PCI, percutaneous coronary intervention; STEMI, ST-segment elevation myocardial infarction; WBC, white blood cell (Count).

a Not pooled due to extreme heterogeneity/unstable estimate.

#### 3.4.1 Demographic factors

Pooled results from nine studies indicated that advanced age was a significant predictor of cardiac rupture, with a combined odds ratio (OR) of 2.26 (95% CI: 1.29–3.95). However, substantial heterogeneity was observed among these studies (*I*^2^ = 95%, *τ*^2^ = 0.392). Female sex was associated with a significantly higher risk of cardiac rupture compared to male sex. The meta-analysis of five studies yielded a pooled OR of 2.43 (95% CI: 1.92–3.07), with no significant heterogeneity (*I*^2^ = 0%).

#### 3.4.2 Clinical presentation and status on admission

A higher Killip grade (III–IV vs. I–II) was a strong predictor, with a pooled OR of 3.58 (95% CI: 3.46–3.71) from three studies. The analysis showed no heterogeneity (*I*^2^ = 0%). Elevated heart rate on admission was significantly associated with increased risk. The combined OR from three studies was 2.29 (95% CI: 1.72–3.06), with no heterogeneity (*I*^2^ = 0%). The association between a longer time to hospital admission and cardiac rupture risk was not statistically significant. The pooled OR from three studies was 1.64 (95% CI: 0.45–5.90), albeit with considerable heterogeneity (*I*^2^ = 93%, *τ*^2^ = 0.226).

#### 3.4.3 Biomarkers and imaging findings

A lower LVEF was a significant predictor of cardiac rupture. The meta-analysis of four studies produced a pooled OR of 1.46 (95% CI: 1.28–1.67) with no heterogeneity (*I*^2^ = 0%). A high WBC count on admission showed a positive but non-significant association with cardiac rupture risk (OR = 1.47, 95% CI: 0.46–4.71). The analysis was marked by high heterogeneity (*I*^2^ = 95%, *τ*^2^ = 0.206). Elevated CRP levels were reported as a risk factor in two studies. However, due to extreme statistical heterogeneity (*I*^2^ = 94%) and an event rate leading to computationally unstable effect estimates, a reliable pooled odds ratio could not be derived. Therefore, these studies were not included in the quantitative meta-analysis. The individual study results suggested a positive association, but this finding must be interpreted with great caution. The presence of pericardial effusion on imaging was examined in two studies. Similar to CRP, quantitative pooling was precluded by substantial heterogeneity (*I*^2^ = 65%) and unstable effect estimates with an extremely wide confidence interval. Individually, both studies reported a strong positive association, indicating pericardial effusion may be a critical imaging marker, but a precise summary estimate could not be established.

#### 3.4.4 Treatment factor

Undergoing emergency percutaneous coronary intervention (PCI) was identified as a significant protective factor against cardiac rupture. The meta-analysis of six studies showed a pooled OR of 0.37 (95% CI: 0.20–0.70), indicating that patients receiving emergency PCI had a 63% reduced odds of rupture. Heterogeneity was low (*I*^2^ = 29%).

#### 3.4.5 Subgroup analysis by participant population

Subgroup analysis based on the participant population (all-comer AMI vs. STEMI-only) was performed to explore potential sources of heterogeneity and to assess the consistency of the associations. The detailed results are presented in Table 4.

The substantial heterogeneity observed in the overall analysis (*I*^2^ = 95%) appeared to be driven by differences in participant populations. The effect of advanced age was markedly stronger and more precise in studies enrolling all AMI patients (OR = 4.75, 95% CI: 0.81–27.76; *I*^2^ = 41%) compared to those restricted to STEMI patients (OR = 1.77, 95% CI: 0.96–3.26; *I*^2^ = 96%), although both estimates retained wide confidence intervals. The protective effect of a higher LVEF was consistent across subgroups but was stronger and highly precise in all-AMI populations (OR = 1.63, 95% CI: 1.52–1.73) than in STEMI-only populations, where the association was not statistically significant (OR = 1.42, 95% CI: 0.71–2.84). The overall protective effect of emergency PCI was evident (OR = 0.37). The point estimates within subgroups suggested a potentially stronger protective effect in STEMI-only cohorts (OR = 0.19) than in all-AMI cohorts (OR = 0.39), though the confidence intervals for both were wide and included the null value. The increased risk associated with female gender was robust and consistent across both all-AMI (OR = 2.75, 95% CI: 1.40–5.41) and STEMI-only (OR = 2.37, 95% CI: 1.40–3.99) populations, with no heterogeneity in either subgroup.

Several factors were exclusively analyzed in one specific population. Killip grade, time-to-hospital admission, and white blood cell count were only evaluated in STEMI populations, with Killip grade being a particularly strong and precise predictor. Conversely, CRP and pericardial effusion were only analyzed in all-AMI studies, showing large but highly imprecise effect estimates.

### 3.5 Model performance

The C-statistic (AUC-ROC) was the primary measure for assessing model performance, documented in all included studies. The sensitivity and specificity were also widely utilized, featured in 60% of the studies. Net reclassification index (NRI) and discrimination enhancement measure (IDI) were less prevalent, reported in only 20% of the analyses. For evaluating calibration, the Hosmer–Lemeshow goodness-of-fit test was the most commonly applied technique (80%). A summary of the performance assessment criteria for each investigation is provided in Tables 3, 5.

**Table 5:** Comparison of model performance using discrimination and reclassification metrics.

Studies	Best performance model	Sensitivity (%)	Specificity (%)	NRI	IDI	AUC
Abulimiti et al. (2022) (14)	LR	94.6	81.1	NI	NI	0.930
Bai (2024) (15)	LR	94.5	90.3	NI	NI	0.907
Fu et al. (2019) (6)	LR	NI	NI	NI	NI	0.843
Luo et al. (2022) (17)	LR	84.4	89.8	NI	NI	0.906
Qian et al. (2017) (13)	LR	NI	NI	NI	NI	0.780
Wu et al. (2024) (19)	LR	90.4	87.6	NI	NI	0.947
Wubuli et al. (2020) (16)	LR	NI	NI	NI	NI	0.771
Yan (2021) (20)	LR	86.8	74.8	0.356	0.054	0.829
Yisimitila et al. (2023) (18)	LR	NI	NI	<0.001	<0.001	0.971
Zhang et al. (2024) (21)	LR	90.7	84.4	NI	NI	0.924

AUC, area under the curve; IDI, integrated discrimination improvement; LR, logistic regression; NI, not provided; NRI, net reclassification improvement.

**Figure 2:**
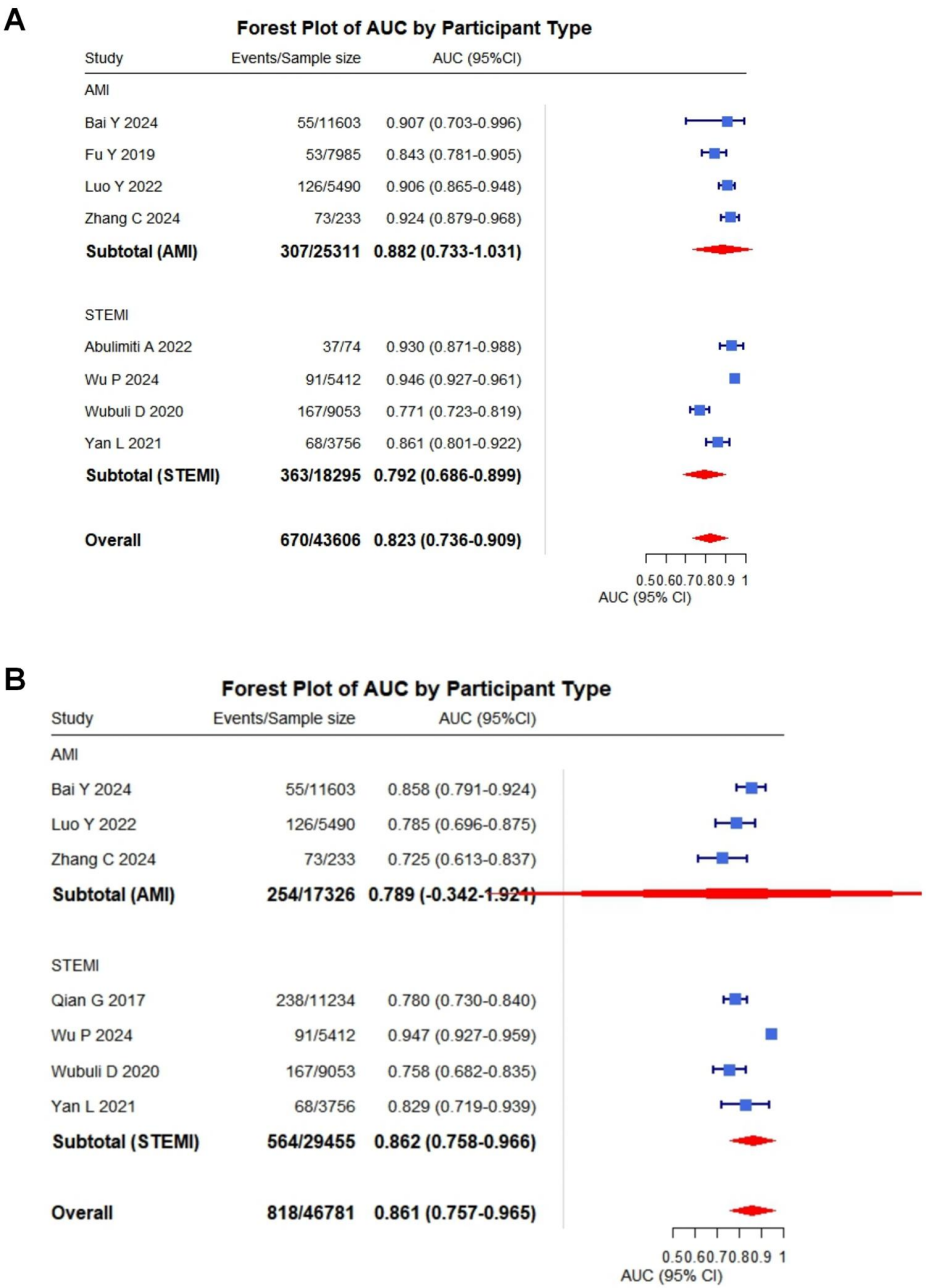
Pooled C-statistics for the prediction models of cardiac rupture post-AMI. **(A)** Forest plot of the C-statistic in model development datasets. **(B)** Forest plot of the C-statistic in independent validation datasets.

Of the 10 included studies, 1 were excluded from quantitative synthesis due to insufficient reporting on model development, leaving 9 studies eligible for meta-analysis. Overall, the meta-analysis demonstrated that these models exhibited moderate to good discrimination in predicting cardiac rupture after AMI. A total of eight studies reported C-statistics for the development sets (range: 0.77–0.95) (6, 14–17, 19–21). The pooled C-statistic was 0.82 (95% CI: 0.74–0.91). Seven studies reported the C-statistics validated on independent datasets (range: 0.73–0.95) (13, 15–17, 19–21). The pooled C-statistic was 0.86 (95% CI: 0.76–0.97) (Figures 2A,B). To provide a more comprehensive overview, we conducted an integrative analysis incorporating nine studies (Supplementary Figure S2A), which yielded a pooled C-statistic of 0.83 (95% CI: 0.78–0.89). However, substantial heterogeneity was observed (*I*^2^ = 88%, *p* < 0.001), prompting subgroup analyses to investigate potential sources of variability in cardiac rupture post infarction (see Supplementary Figures S2B–L; Table 6).

**Table 6:** Subgroup analyses of model performance based on AUC.

Category	Subgroups	No studies	Heterogeneity test		Effects models	Meta-analysis
			*I*^2^ (%)	*P*		*OR* (95%*CI*)
Publication period	2017–2021	4	17	0.31	fixed effects models	0.80 (0.77, 0.84)
	2022–2024	5	82	<0.001	random effects models	0.87 (0.80, 0.94)
Study design	Retrospective study	8	84	<0.001	random effects models	0.84 (0.79, 0.90)
	Retrospective study and prospective study	1	Not applicable			
Participants population	AMI	4	27	0.25	fixed effects models	0.83 (0.79, 0.87)
	STEMI	5	91	<0.001	random effects models	0.85 (0.77, 0.94)
Sample size	≥1,000	7	90	<0.001	random effects models	0.83 (0.76, 0.90)
	<1,000	2	86	0.007	random effects models	0.83 (0.65, 1.06)
Outcomes	CR	7	90	<0.001	random effects models	0.84 (0.78, 0.91)
	FWR	2	68	0.08	random effects models	0.80 (0.68, 0.94)
Validation approach	No internal validation study	3	73	0.03	random effects models	0.86 (0.78, 0.94)
	Internal validation study	6	91	<0.001	random effects models	0.82 (0.74, 0.91)

AMI, acute myocardial infarction; CI, confidence interval; CR, cardiac rupture; FWR, free-wall rupture; *I*², I-squared; No., number; OR, odds ratio; STEMI, ST-elevation myocardial infarction.

#### 3.5.1 Subgroup analysis by publication period

Subgroup analysis based on publication period demonstrated a clear temporal trend in model performance and heterogeneity. For studies published from 2017 to 2021 (*n* = 4), the pooled C-statistic was 0.80 (95% CI: 0.77–0.84) with low heterogeneity (*I*^2^ = 17%, *p* = 0.31). In contrast, studies published between 2022 and 2024 (*n* = 5) exhibited a higher discriminative performance, with a pooled AUC of 0.87 (95% CI: 0.80–0.94), albeit with substantial heterogeneity (*I*^2^ = 82%, *p* < 0.001).

#### 3.5.2 Subgroup analysis by study design

Eight studies employed a retrospective design, yielding a pooled AUC of 0.84 (95% CI: 0.79–0.90) with high heterogeneity (*I*^2^ = 84%, *p* < 0.001). Only one study utilized a combined retrospective and prospective design; therefore, a comparative quantitative analysis for this subgroup was not feasible.

#### 3.5.3 Subgroup analysis by participants population

When stratified by participant population, studies including mixed AMI patients (*n* = 4) showed a pooled AUC of 0.83 (95% CI: 0.79–0.87) with low heterogeneity (*I*^2^ = 27%, *p* = 0.25). Conversely, studies restricted to STEMI patients (*n* = 5) demonstrated a similar pooled AUC of 0.85 (95% CI: 0.77–0.94) but with considerable heterogeneity (*I*^2^ = 91%, *p* < 0.001).

#### 3.5.4 Subgroup analysis by sample size

Analysis by sample size revealed that larger studies (≥1,000 patients, *n* = 7) had a pooled AUC of 0.83 (95% CI: 0.76–0.90) and high heterogeneity (*I*^2^ = 90%, *p* < 0.001). Smaller studies (<1,000 patients, *n* = 2) showed a comparable point estimate for AUC (0.83, 95% CI: 0.65–1.06) with significant heterogeneity (*I*^2^ = 86%, *p* = 0.007), although the wider confidence interval indicates greater imprecision.

#### 3.5.5 Subgroup analysis by outcome

For studies predicting general cardiac rupture (*n* = 7), the pooled AUC was 0.84 (95% CI: 0.78–0.91) with high heterogeneity (*I*^2^ = 90%, *p* < 0.001). Models specifically predicting FWR (*n* = 2) demonstrated a pooled AUC of 0.80 (95% CI: 0.68–0.94) with moderate, non-significant heterogeneity (*I*^2^ = 68%, *p* = 0.08).

#### 3.5.6 Subgroup analysis by validation approach

Subgroup analysis based on validation approach indicated that studies without internal validation (*n* = 3) had a pooled AUC of 0.86 (95% CI: 0.78–0.94) with moderate heterogeneity (*I*^2^ = 73%, *p* = 0.03). Studies that performed internal validation (*n* = 6) showed a slightly lower pooled AUC of 0.82 (95% CI: 0.74–0.91), accompanied by high heterogeneity (*I*^2^ = 91%, *p* < 0.001). Detailed results of all subgroup analyses are summarized in Table 6.

#### 3.5.7 Publication bias assessment

Visual inspection of the funnel plot suggested minor asymmetry (Supplementary Figure S2M). However, Egger's regression test did not provide statistical evidence for significant publication bias (intercept = 1.106, 95% CI: 0.542–1.670, *p* = 0.418). Therefore, the risk of substantial small-study effects or reporting bias was considered low.

### 3.6 Studies comparing models

Four studies (6, 13, 16, 20) compared two risk scores or models in the same population.

#### 3.6.1 GRACE

Although the GRACE score was not originally developed to predict cardiac rupture, its evaluation in two included studies (6, 13) demonstrated moderate discriminative ability, with reported C-statistics ranging from 0.716 to 0.73. The incidence of CR in these studies varied between 0.6% and 2.12%. The GRACE score's predictive utility for CR may stem from its incorporation of several high-risk features shared with CR pathogenesis, such as advanced age, elevated Killip class, and ST-segment deviations—all of which are associated with larger infarct size and left ventricular dysfunction, key contributors to CR. Additionally, the GRACE score's widespread clinical adoption for acute coronary syndrome risk stratification offers practical advantages, as its integration into existing workflows would require minimal additional effort if validated for CR prediction. However, the modest discrimination (AUC < 0.75) suggests that while the GRACE score captures some risk overlap, it lacks optimal precision as a standalone tool for CR prediction. This limitation likely arises from the absence of CR-specific predictors in its variables. Further research is needed to determine whether combining GRACE with CR-specific factors could enhance performance.

#### 3.6.2 Qian G et al. Model

Among the three studies (13, 16, 20) included in this systematic review that evaluated the risk prediction model developed by Qian et al., the reported incidence of CR post infarction ranged from 1.81% to 2.12%. The discriminative ability of the model, as measured by the C-statistic, varied across studies, with values ranging from 0.68 to 0.78, indicating moderate predictive performance. In the study by Yan et al., the model demonstrated a sensitivity of 86.8% and a specificity of 74.8%, suggesting reasonable accuracy in identifying both true-positive and true-negative cases. Calibration was assessed using the Hosmer-Lemeshow goodness-of-fit test. In two studies, the model showed good calibration: Yan et al. reported a chi-square statistic of 4.949 (*P* = 0.763), indicating no significant deviation between predicted and observed risks. Similarly, Wubuli et al. found no evidence of poor fit (*P* = 0.669). Overall, the Qian et al. model exhibited moderate discrimination and good calibration in predicting cardiac rupture risk post-AMI across the included studies. However, further external validation is warranted to confirm its generalizability.

### 3.7 Risk of bias and applicability assessment

#### 3.7.1 Participants, predictors, outcomes

All 10 included studies failed to report whether the adjudication of cardiac rupture was performed blindly without knowledge of predictor information. Specifically, no studies reported whether the diagnosis of cardiac rupture was independently verified by assessors blinded to the predictive factors, nor did any study clarify whether the diagnostic methods employed were sufficient to avoid the influence of predictive factor information. Although cardiac rupture is primarily diagnosed through objective examinations (e.g., echocardiography, CT, or autopsy), the diagnosis in early-stage or atypical cases may still be subject to clinical judgment. This reporting limitation hinders accurate assessment of the risk of detection bias. Only 2 out of 10 studies explicitly documented the time interval from MI symptom onset to cardiac rupture occurrence (19, 20). The remaining 8 studies failed to specify the temporal relationship between predictor measurement and outcome onset, making it difficult to evaluate the potential impact of timing on model performance. Notably, both studies reporting time intervals indicated that cardiac rupture predominantly occurred within 7 days post-MI, suggesting that predictor measurement might need to target this critical window. Three out of ten studies did not explicitly report the inclusion and exclusion criteria for participants (6, 16, 20). The remaining seven studies provided detailed descriptions of the screening process for the study population. All of them adopted internationally recognized diagnostic criteria for myocardial infarction (e.g., ESC/ACC guidelines) and excluded patients with conditions that could potentially confound the assessment of cardiac rupture, such as severe valvular disease or traumatic cardiac injury. A detailed PROBAST-based assessment of population, predictors, and outcomes is presented in Supplementary Table S8.

#### 3.7.2 Analysis-model development studies

None of the 10 included studies met the recommended standard of at least 10 outcome events per predictor variable (EPV ≥ 10) as per the rule of thumb. The number of predictor variables across the studies ranged from 20 to 48, whereas the corresponding number of cardiac rupture events was only 37–238, resulting in EPV ratios between 1.0 and 9.2 (see Table 1 for details). This methodological limitation may significantly compromise the precision of model parameter estimates and increase the risk of overfitting. None of the 10 included predictive modeling studies explicitly reported their approaches for handling common complexities in clinical data. Specifically: (i) no study mentioned strategies for addressing missing data (e.g., multiple imputation); (ii) none described methods for handling time-dependent variables (e.g., dynamic post-admission indicator changes); (iii) there was a lack of exploration of nonlinear relationships or interactions among variables; and (iv) no studies reported adjustment measures for clustering effects (e.g., multicenter data) or competing risks (e.g., non-cardiac rupture mortality). Nine out of the ten included studies exhibited methodological flaws in predictor selection. All these studies screened potential predictors based solely on univariate analysis results (e.g., variables with *p*-value <0.05), without considering multivariate synergistic effects or clinical relevance. Only one study (19) employed regularization methods such as LASSO regression for variable selection, thereby reducing overfitting risks. This prevalent use of univariate screening may lead to: (i) omission of important predictors that fail to reach significance thresholds in univariate analysis; (ii) inclusion of spurious association variables (particularly in small-sample studies); and (iii) overestimation of the final model's predictive performance. Eight studies determined the cutoff values for continuous variables based on their own datasets or did not employ clinically recognized standard thresholds. One study (19) avoided dichotomization of continuous variables, thereby reducing the risk of such bias. This approach may lead to overfitting of the predictive model to the original dataset, compromising its generalizability. Four out of ten studies failed to report any internal validation methods. These studies (6, 14, 17, 18) only presented performance metrics during model development (such as C-statistic or calibration), but lacked correction for potential optimism bias in the derivation sample. The remaining 6 studies employed methods such as dataset splitting (*n* = 4), bootstrap resampling (*n* = 1) or temporal validation (*n* = 1); however, only 2 study (14, 20) comprehensively reported the differences between corrected performance metrics and original estimates. Two studies (6, 15) (20%) directly excluded participants with missing data without employing any imputation methods, thus being rated as high risk of bias. The remaining eight studies did not explicitly report the presence or handling of missing data, resulting in uncertainty in the assessment of this domain. These limitations in missing data handling may compromise the accuracy and generalizability of the model's predictive performance, particularly in real-world clinical settings where missing data are prevalent. The detailed PROBAST assessment of analysis-related bias is provided in Supplementary Table S8 (analysis domain) and Supplementary Table S9.

#### 3.7.3 Analysis-model evaluation studies

**Figure 3:**
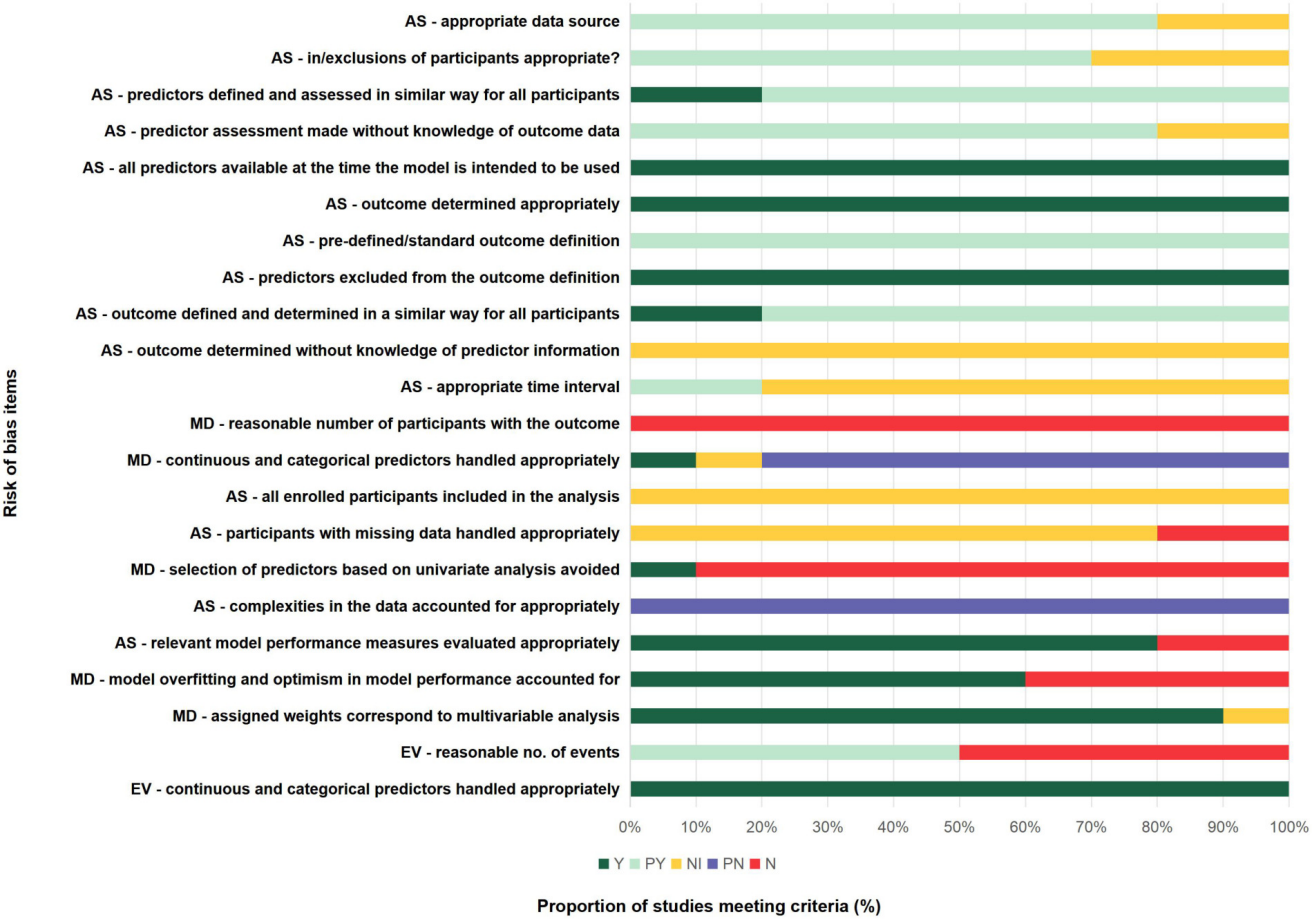
Risk of bias summary. Chart shows percentage of study cohorts meeting/not meeting criteria: AS, all studies (10 study cohorts); EV, external validation studies (4 study cohorts); MD, model development studies (10 study cohorts); N, no or insufficient information; PN, probably no; PY, probably yes, Y, yes.

Two of the four external validation studies were judged to be at high risk of bias due to having fewer than 100 outcome events or a total sample size below 1,000 (17, 20), potentially limiting the reliability of model performance estimates. The remaining two studies met the recommended criteria and were judged to be at low risk of bias (6, 13). The models were applied exactly as originally developed in all four external validation studies, with the same dichotomization and cut-offs as in the development studies, and the original prediction equations were applied exactly as created. This suggests that the methodological rigor in model application was maintained across these studies, reducing concerns about inappropriate handling of predictors. A comprehensive risk of bias appraisal adapted from PROBAST is summarized in Supplementary Table S9, while Figure 3 provides an overall visual summary (all studies, external validation studies, and model development studies).

#### 3.7.4 Adherence to reporting standards

**Figure 4:**
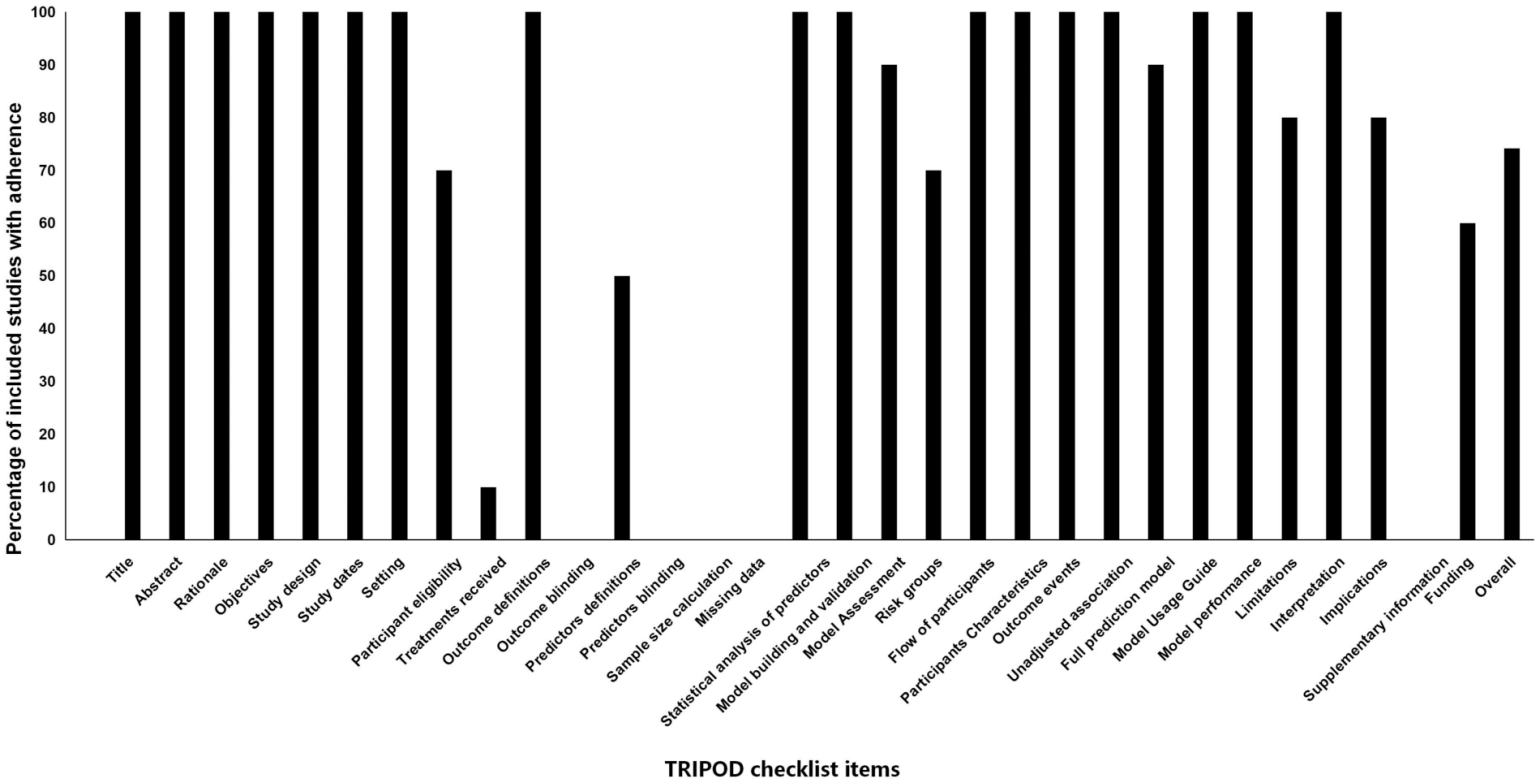
Adherence of included studies to transparent reporting of a multivariable preduction model for individual prognosis or diagnosis (TRIPOD) assessment.

Assessment of reporting quality using the TRIPOD checklist revealed an overall adherence rate of 74.2%, with substantial variation across checklist items. Essential elements including title, abstract, rationale, and objectives were consistently reported across all studies (100%). Similarly, methodological components such as study design, setting, and outcome definitions demonstrated complete adherence (100%). However, critical methodological details showed poor reporting: only 10% of studies described treatments received, and none reported outcome blinding or predictor blinding (0%). Model development and validation elements were generally well-reported, with model development/validation, statistical analysis of predictors, and model performance showing complete adherence (100%). However, pre-modeling considerations were frequently neglected, including sample size calculations and handling of missing data (0%). Clinical implementation components exhibited moderate reporting: risk groups (70%), model usage guidance (100%), and model performance (100%) were reasonably well-reported, while only 60% of studies disclosed funding sources. A detailed item-by-item adherence to the TRIPOD checklist is illustrated in Figure 4.

## 4 Discussion

### 4.1 Principal findings

**Figure 5:**
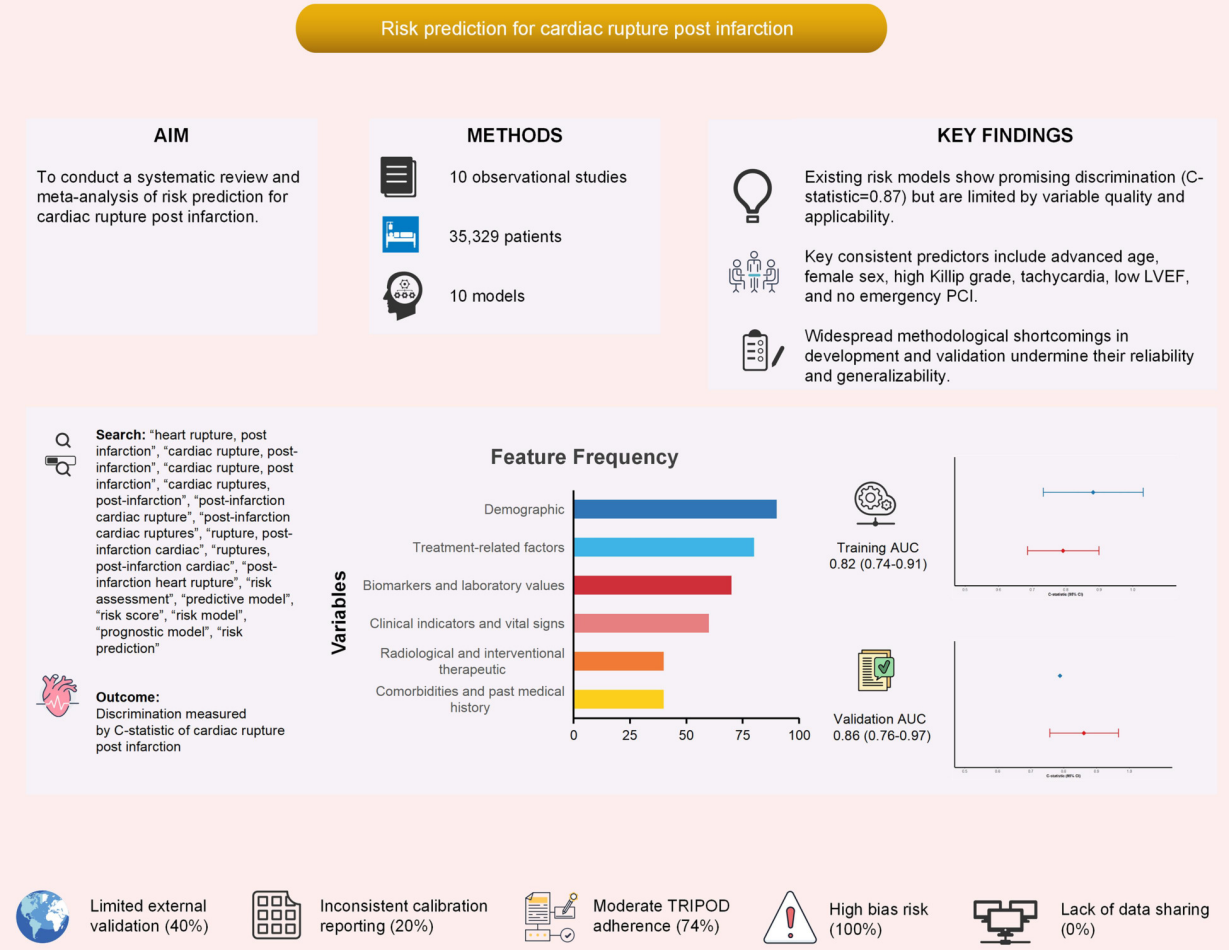
Visual summary of the main findings in our study. Icons from WPS Office (https://www.wps.com/).

This study represents a systematic review and meta-analysis synthesizing evidence on risk prediction models for CR after AMI. From 619 screened records, 10 studies were included, encompassing logistic regression–based models with variable methodological rigor and limited external validation. Our meta-analysis identified several consistently significant predictors of CR: advanced age, female sex, higher Killip grade, elevated heart rate, reduced LVEF, and the absence of emergency PCI. Among these, Killip grade and female sex demonstrated the strongest associations with rupture risk. The overall pooled discriminative performance of CR prediction models was robust (C-statistic 0.83, 95% CI: 0.78–0.89), though substantial heterogeneity was observed. Quality appraisal indicated methodological deficiencies in predictor selection, handling of missing data, and internal validation, underscoring the need for more rigorous model development and evaluation (Figure 5). It is crucial to interpret the high pooled discriminative performance with caution. As assessed by the PROBAST tool, all included studies exhibited significant methodological limitations that predispose to model overfitting and optimism. Most critically, the EPV ratio across studies ranged from only 1.0 to 9.2, far below the recommended threshold of ≥10 to ensure reliable and generalizable estimates. This low EPV, combined with a predominant reliance on univariate screening for predictor selection and inadequate handling of missing data, strongly suggests that the reported C-statistics are likely optimistic estimates of performance on development datasets rather than indicative of true predictive accuracy in new populations. Therefore, the true external validity and clinical utility of these models remain uncertain.

### 4.2 Interpretation in the context of existing evidence

Our findings corroborate long-standing clinical observations that advanced age and female sex are strong predictors of CR. Aging myocardium is characterized by diminished collagen content and impaired healing capacity, which may predispose to rupture (22, 23). Similarly, female patients often present later and exhibit microvascular dysfunction and smaller body size, factors that may exacerbate wall stress after infarction (24–26). Elevated Killip grade and tachycardia reflect hemodynamic instability and larger infarct size, both biologically plausible determinants of rupture (24). Reduced LVEF highlights impaired ventricular integrity and has been repeatedly associated with structural complications in prior cohort studies (24, 25, 27). The protective role of emergency PCI aligns with evidence that timely reperfusion limits infarct expansion and preserves myocardial wall stability (26, 28).

In terms of risk models, our results show that general scores such as GRACE, while incorporating some overlapping variables, provide only modest discrimination (AUC <0.75) for CR, reflecting their design for broader acute coronary syndrome prognosis rather than rupture-specific risk. In contrast, models tailored for CR, such as Qian et al.'s, demonstrated better calibration but only moderate discrimination, indicating that inclusion of CR-specific predictors is crucial. Collectively, our analysis supports the development of a dedicated CR risk model that integrates both traditional clinical predictors and novel biomarkers. Thus, while these tailored models show conceptually improved design, their reported superior performance metrics, like the pooled C-statistic in our meta-analysis, must be viewed as provisional and likely inflated due to the aforementioned methodological shortcomings.

### 4.3 Clinical implications and utility

The predictors identified in this review can serve as practical red flags for frontline clinicians, enabling rapid recognition of high-risk patients during the vulnerable early post-infarction period. Risk stratification based on age, sex, Killip class, heart rate, and LVEF could guide intensified monitoring in coronary care units and more cautious use of therapies that may elevate rupture risk, such as aggressive thrombolysis or high-dose anticoagulation (29). Emergency PCI emerges not only as a therapeutic cornerstone for infarct salvage but also as a preventive measure against rupture, reinforcing its prioritization in treatment pathways (26, 28). Despite promising performance metrics, no existing model is currently ready for routine clinical deployment due to lack of large-scale external validation. Nevertheless, the pooled evidence presented here provides a solid foundation for constructing a standardized and clinically applicable CR-specific prediction tool.

### 4.4 Methodological considerations

The ostensibly encouraging performance metrics, including our meta-analytic summary C-statistic, are substantially undermined by critical methodological flaws that introduce significant optimism bias. Most studies failed to meet the recommended threshold of ≥10 events per predictor variable, raising substantial concerns of overfitting (30). Predictor selection was predominantly based on univariate screening rather than multivariable or clinical relevance-driven strategies, which may result in omission of important predictors and spurious associations (31). Few studies employed advanced techniques such as penalized regression or machine learning, which could better accommodate complex interactions and nonlinearities (32). Furthermore, missing data were either ignored or handled through complete-case analysis, despite the availability of multiple imputation methods. External validation was infrequent, and where performed, often underpowered due to small sample sizes or limited outcome events (33–36). These deficiencies collectively limit generalizability and may explain the high heterogeneity observed in our pooled analyses.

### 4.5 Strengths and limitations of the review

The strengths of our review include the use of rigorous methodological frameworks (PRISMA, CHARMS, TRIPOD, and PROBAST), comprehensive literature search across English and Chinese databases, and quantitative synthesis of predictors and model performance. However, several limitations must be acknowledged. First, the number of included studies was relatively small, and their heterogeneity in design, populations, and outcome definitions constrained the scope of pooled analyses. Second, and most critically regarding the interpretation of model performance, our quantitative synthesis is fundamentally limited by the poor methodological quality of the primary studies, as identified by PROBAST. The universal issue of a low EPV ratio (range: 1.0–9.2) and the widespread use of suboptimal predictor selection strategies mean that the primary studies' reported C-statistics—and consequently our pooled estimate of 0.83—are almost certainly subject to severe overfitting and optimism bias. This estimate should therefore be interpreted not as a reliable indicator of future model performance but as an upper bound of discrimination achievable on development datasets under ideal, non-generalizable conditions. The true prospective discriminatory ability of these models in external, real-world populations is likely to be substantially lower. Third, potential publication bias cannot be entirely excluded, despite negative findings from Egger's test. Fourth, our meta-analysis of certain predictors, notably CRP and pericardial effusion, was limited by extreme statistical heterogeneity and unstable effect estimates, preventing meaningful quantitative pooling. This instability likely stems from the low event rate of cardiac rupture, small number of studies investigating these specific factors, and potential differences in measurement timing, assay methods (for CRP), or imaging protocols. While these factors were individually reported as significant predictors in primary studies, their exact effect size remains uncertain, highlighting the need for standardized reporting and larger-scale studies to confirm their prognostic utility. Fifth, most included studies were retrospective, limiting causal inference and increasing susceptibility to bias. Sixth, we only included studies published in English and Chinese, which may have introduced language bias. Finally, although we identified robust predictors, our ability to determine their incremental value beyond established risk scores was limited by incomplete reporting of net reclassification or decision-curve analyses.

### 4.6 Future directions

Our findings underscore several priorities for future research. First and foremost, future model development studies must adhere to fundamental methodological standards to mitigate overfitting. This includes ensuring an adequate EPV ratio (≥10–20), employing clinical knowledge and penalized regression techniques alongside data-driven selection for predictors, and utilizing appropriate internal validation methods to correct for optimism in performance estimates. Until these standards are met, any reported high discrimination should be considered preliminary. Second, adequately powered, prospective, multicenter studies are needed to validate and recalibrate existing models across diverse populations and clinical settings. Third, the incorporation of dynamic and time-dependent variables such as temporal changes in hemodynamics, biomarkers, or imaging parameters may enhance predictive accuracy during the critical early post-infarction period (37, 38). Fourth, advanced statistical and machine learning methods should be applied to optimize variable selection, reduce overfitting, and capture nonlinear interactions (39, 40). Fifth, standardized reporting of prediction model studies, following TRIPOD guidelines, must be enforced to improve transparency and reproducibility (41). Finally, integration of CR-specific models into electronic health record systems and real-time clinical decision support tools should be explored to facilitate practical implementation.

## 5 Conclusion

In conclusion, this review identifies consistent clinical predictors and suggests that existing prediction models demonstrate promising but likely optimistic discriminatory performance in their development phases. However, due to pervasive methodological limitations, particularly very low events-per-variable ratios, the current evidence is insufficient to recommend any model for clinical use. While several risk prediction models for cardiac rupture after AMI demonstrate promising discrimination, methodological weaknesses and limited external validation currently preclude their widespread adoption. Robust predictors such as age, female sex, Killip grade, LVEF, and PCI status should form the foundation of future consensus-based models. Future research must prioritize methodological rigor, external validation, and assessment of clinical impact. There is an urgent need for large-scale, prospective, and externally validated studies to translate these potential predictors into reliable tools for clinical risk stratification and targeted prevention of this fatal complication.

## Data Availability

The original contributions presented in the study are included in the article/Supplementary Material, further inquiries can be directed to the corresponding authors.
