# Supplementary material for: Risk prediction models for cardiac rupture after acute myocardial infarction: a systematic review and meta-analysis
Source: Front Cardiovasc Med. 2026 Feb 11;13:1721103. doi: 10.3389/fcvm.2026.1721103 (PMC12933645; doi:10.3389/fcvm.2026.1721103)
Supplement: Supplementary file 3 [file Table3.docx]

**Supplementary Table S3. TRIPOD Checklist**

| **Section/Topic Item Checklist Item Information in this study** | | | |
| --- | --- | --- | --- |
| **Title and abstract** | | | |
| Title | 1 | Identify the study as the development/validation/systematic review/meta-analysis of a prediction model | Systematic review and meta-analysis of risk prediction models for cardiac rupture after AMI |
| Abstract | 2 | Provide background, objectives, methods, results, and conclusions | Included: background (clinical importance of AMI-CR), objectives (evaluate model performance), methods (systematic review + meta-analysis), results (AUC, risk factors), conclusions |
| **Introduction** | | | |
| Background and objectives | 3 | Explain rationale, clinical context, and objectives | Cardiac rupture is a severe complication of AMI; objective: to systematically review risk prediction models and summarize prognostic factors |
| **Methods** | | | |
| Source of data | 4 | Data sources, search dates | Multiple databases, searched up to July 31, 2025; language restricted to English and Chinese |
| Participants | 5 | Inclusion/exclusion criteria, study setting | Inclusion: patients with AMI (including STEMI); Exclusion: non-AMI-related rupture (traumatic/iatrogenic); Settings: emergency, cardiology, and ICU (all included studies from China) |
| Outcome | 6 | Clearly define predicted outcomes | Cardiac rupture (CR/FWR), defined according to ACC/ESC criteria |
| Predictors | 7 | Candidate predictors and measurement | Age, sex, Killip class, LVEF, heart rate, CRP, WBC, PCI status, time-to-hospital admission, comorbidities (Tables 3–4) |
| Sample size | 8 | Report number of participants and events | Study sample sizes ranged 74–11,603; event numbers 37–238 (Table 2) |
| Missing data | 9 | Describe handling of missing data | Approaches varied: direct deletion, not reported, or not applicable (Table 3) |
| Statistical analysis methods | 10 | Model development methods, predictor selection, validation, calibration | Logistic regression (mostly univariate screening, stepwise selection, some LASSO); Calibration: Hosmer–Lemeshow test; Validation: temporal, geographic, bootstrap (Table 3) |
| Model Development | 11 | Method for predictor inclusion | Univariate logistic regression, stepwise regression, or LASSO (Wu P 2024) |
| Model Performance | 12 | Report discrimination, calibration, sensitivity, specificity | AUC ranged 0.771–0.971; some studies reported sensitivity/specificity (e.g., Abulimiti A 2022: 94.6%/81.1%) (Table 5) |
| Model Presentation | 13 | Presentation of the final model (e.g., score, nomogram) | Most models presented as risk scores; nomograms reported in Wu P 2024, Yan L 2021, Yisimitila T 2023 (Table 3) |
| **Results** | | | |
| Participants | 14 | Numbers, baseline characteristics | Ten studies included, all conducted in China, covering 2010–2023 (Table 2) |
| Predictors | 15 | Report final predictors included in models | Age, sex, Killip class, LVEF, heart rate, CRP, WBC, PCI status, admission delay (Tables 3–4) |
| Model performance | 16 | Report measures of model performance | Good to excellent discrimination (AUC 0.771–0.971); calibration reported in some models; external validation limited |
| Meta-analysis | 17 | Pooled findings | Strong risk factors: female sex (OR=2.43), higher Killip class (OR=3.58), reduced LVEF (OR=1.46), elevated HR (OR=2.29), high CRP (OR≈5.67) (Table 4) |
| Subgroup Analysis | 18 | Subgroups and heterogeneity | Publication period (2017–2021 vs 2022–2024), population (AMI vs STEMI), outcomes (CR vs FWR), sample size (≥1000 vs <1000), validation approach (internal vs none) (Table 6) |
| **Discussion** | | | |
| Limitations | 19 | Study limitations | All studies conducted in China; small event numbers in some cohorts; lack of external validation; missing data handling often unclear |
| Implications | 20 | Clinical and research implications | Findings highlight essential predictors for future multicenter, international models to improve generalizability and clinical utility |
| **Other information** | | | |
| Supplementary information | 21 | Funding, conflicts of interest, supplementary materials | To be reported in final manuscript; Tables 1–6 can serve as supplementary material |
