# Supplementary material for: Risk prediction models for cardiac rupture after acute myocardial infarction: a systematic review and meta-analysis
Source: Front Cardiovasc Med. 2026 Feb 11;13:1721103. doi: 10.3389/fcvm.2026.1721103 (PMC12933645; doi:10.3389/fcvm.2026.1721103)
Supplement: Supplementary file 4 [file Table4.docx]

**Supplementary Table S4.** **Complete list of search terms**

| Search Terms |
| --- |
| (“Heart Rupture, Post Infarction”[MESH] OR “Cardiac Rupture, Post-Infarction”[Title/Abstract] OR “Cardiac Rupture, Post Infarction”[Title/Abstract] OR “Cardiac Ruptures, Post-Infarction”[Title/Abstract] OR “Post-Infarction Cardiac Rupture”[Title/Abstract] OR “Post-Infarction Cardiac Ruptures”[Title/Abstract] OR “Rupture, Post-Infarction Cardiac”[Title/Abstract] OR “Ruptures, Post-Infarction Cardiac”[Title/Abstract] OR “Post-Infarction Heart Rupture”[Title/Abstract] OR “Heart Ruptures, Post-Infarction”[Title/Abstract] OR “Post Infarction Heart Rupture”[Title/Abstract] OR “Post-Infarction Heart Ruptures”[Title/Abstract] OR “Rupture, Post-Infarction Heart”[Title/Abstract] OR “Ruptures, Post-Infarction Heart”[Title/Abstract])  AND  (“risk assessment”[MESH] OR “risk assessment”[Title/Abstract] OR predict*[Title/Abstract] OR “predictive model”[Title/Abstract] OR “predicting model”[Title/Abstract] OR “risk score”[Title/Abstract] OR “risk model”[Title/Abstract] OR “prognostic model”[Title/Abstract]) OR “risk prediction”[Title/Abstract] OR “risk factors*”[Title/Abstract]) |

MESH, Medical Subject Headings

| **Number** | **Search Terms** | **Medline (PubMed)** | **Embase** | **Web of Science** | **Cochrane** | **CNKI** | **Wanfang** |
| --- | --- | --- | --- | --- | --- | --- | --- |
| 1 | ((Heart Rupture, Post Infarction[MeSH Terms]) OR (Cardiac Rupture, Post-Infarction[Title/Abstract]) OR (Cardiac Rupture, Post Infarction[Title/Abstract]) OR (Cardiac Ruptures, Post-Infarction[Title/Abstract]) OR (Post-Infarction Cardiac Rupture[Title/Abstract]) OR (Post-Infarction Cardiac Ruptures[Title/Abstract]) OR (Rupture, Post-Infarction Cardiac[Title/Abstract]) OR (Ruptures, Post-Infarction Cardiac[Title/Abstract]) OR (Post-Infarction Heart Rupture[Title/Abstract]) OR (Heart Ruptures, Post-Infarction[Title/Abstract]) OR (Post Infarction Heart Rupture[Title/Abstract]) OR (Post-Infarction Heart Ruptures[Title/Abstract]) OR (Rupture, Post-Infarction Heart[Title/Abstract]) OR (Ruptures, Post-Infarction Heart[Title/Abstract])) | 2,181 | 815 | 1473 | 249 | 1,763 | 1,998 |
| 2 | ((risk assessment”[MESH Terms] OR (risk assessment[Title/Abstract]) OR predict*[Title/Abstract] OR (predictive model[Title/Abstract]) OR (predicting model[Title/Abstract]) OR (risk score[Title/Abstract]) OR (risk model[Title/Abstract]) OR (prognostic model[Title/Abstract]) OR (risk prediction[Title/Abstract]) OR (risk factor*[Title/Abstract]) OR nomogram*[Title/Abstract])) | 3,349,325 | 27,643 | 58,327 | 265,402 | 1,224,770 | 39,266 |
| 4 | 1 AND 2 | 264 | 93 | 127 | 85 | 37 | 13 |

Total = 619

After removal of duplicates (Endnote) = 582

Full text review = 10
