# Supplementary material for: Risk prediction models for cardiac rupture after acute myocardial infarction: a systematic review and meta-analysis
Source: Front Cardiovasc Med. 2026 Feb 11;13:1721103. doi: 10.3389/fcvm.2026.1721103 (PMC12933645; doi:10.3389/fcvm.2026.1721103)
Supplement: Supplementary file 5 [file Table5.docx]

**Supplementary Table S5. Justification of exclusions at full-text review**

**Prognostic factors studies (n=13)**

[1]Mimo R, Sparacino L, Nicolosi GL, Cervesato E, Wei DR, Grandis U, Lestuzzi C, Rellini GL, Charmet PA, Zanuttini D. Echocardiographic prediction of the final event in patients dying of acute myocardial infarction. Eur Heart J. 1993 Jun;14(6):775-9. doi: 10.1093/eurheartj/14.6.775. PMID: 8325304.

[2]Sobkowicz B, Lenartowska L, Nowak M, Hirnle T, Borys D, Kosicki M, Prajs P, Wrabec K. Trends in the incidence of the free wall cardiac rupture in acute myocardial infarction. observational study: experience of a single center. Rocz Akad Med Bialymst. 2005;50:161-5. PMID: 16358958.

[3]Herlitz J, Samuelsson SO, Richter A, Hjalmarson A. Prediction of rupture in acute myocardial infarction. Clin Cardiol. 1988 Feb;11(2):63-9. doi: 10.1002/clc.4960110203. PMID: 3278820.

[4]Ye L, Bai HM, Jiang D, He B, Wen XS, Ge P, Zhang DY. Combination of eosinophil percentage and high-sensitivity C-reactive protein predicts in-hospital major adverse cardiac events in ST-elevation myocardial infarction patients undergoing primary percutaneous coronary intervention. J Clin Lab Anal. 2020 Sep;34(9):e23367. doi: 10.1002/jcla.23367. Epub 2020 May 22. PMID: 32441412; PMCID: PMC7521303.

[5]Oliva PB, Hammill SC, Edwards WD. Cardiac rupture, a clinically predictable complication of acute myocardial infarction: report of 70 cases with clinicopathologic correlations. J Am Coll Cardiol. 1993 Sep;22(3):720-6. doi: 10.1016/0735-1097(93)90182-z. PMID: 8354804.

[6]Widmer A, Linka AZ, Attenhofer Jost CH, Buergi B, Brunner-La Rocca HP, Salomon F, Seifert B, Jenni R. Mechanical complications after myocardial infarction reliably predicted using C-reactive protein levels and lymphocytopenia. Cardiology. 2003;99(1):25-31. doi: 10.1159/000068448. PMID: 12589119.

[7]Pron PG, Angelino P, Varbella F, Bongioanni S, Masi AS, Iazzolino E, Bonfiglio G, Brusin MC, Mainardi L, Nicastro C, Bouslenko Z, Conte MR. Rottura di cuore nell'infarto miocardico acuto: studio multicentrico osservazionale delle unità coronariche piemontesi [Heart rupture in acute myocardial infarction: multicenter observational study of the coronary unit of Piedmont]. Ital Heart J Suppl. 2002 Feb;3(2):215-20. Italian. Erratum in: Ital Heart J 2002 Jun;3(6 Suppl):677. PMID: 11926028.

[8]Yang T, Chen J, Fu X, Xu C, Liu X, Fu P, Niu T. Predictive Effect of Atypical Right Bundle-Branch Block on In-Hospital Sudden Cardiac Death and Cardiac Rupture and Long-Term Prognosis in Patients With Acute Myocardial Infarction Who Underwent Percutaneous Coronary Intervention. J Am Heart Assoc. 2025 Mar 4;14(5):e038344. doi: 10.1161/JAHA.124.038344. Epub 2025 Feb 26. PMID: 40008523; PMCID: PMC12132683.

[9]Kinoshita T, Asai T, Suzuki T. Hyperlactatemia as a Risk Stratification for Postinfarction Ventricular Septal Rupture. Heart Surg Forum. 2022 May 25;25(3):E345-E352. doi: 10.1532/hsf.4479. PMID: 35787770.

[10]Fan X, Wang S, Liu P, Bai L. Inferior ST-Segment Elevation Can Predict In-Hospital Mortality in Patients with Anterior Myocardial Infarction Complicated by Ventricular Septal Rupture. Dis Markers. 2022 Jul 15;2022:7067420. doi: 10.1155/2022/7067420. PMID: 35872699; PMCID: PMC9307369.

[11]Qian G, Liu HB, Wang JW, Wu C, Chen YD. Risk of cardiac rupture after acute myocardial infarction is related to a risk of hemorrhage. J Zhejiang Univ Sci B. 2013 Aug;14(8):736-42. doi: 10.1631/jzus.B1200306. PMID: 23897793; PMCID: PMC3735974.

[12]Qian G, Wu C, Chen YD, Tu CC, Wang JW, Qian YA. Predictive factors of cardiac rupture in patients with ST-elevation myocardial infarction. J Zhejiang Univ Sci B. 2014 Dec;15(12):1048-54. doi: 10.1631/jzus.B1400095. PMID: 25471834; PMCID: PMC4265559.

[13]Sulzgruber P, El-Hamid F, Koller L, Forster S, Goliasch G, Wojta J, Niessner A. Long-term outcome and risk prediction in patients suffering acute myocardial infarction complicated by post-infarction cardiac rupture. Int J Cardiol. 2017 Jan 15;227:399-403. doi: 10.1016/j.ijcard.2016.11.037. Epub 2016 Nov 7. PMID: 27847155.

**Ineligible study outcome** **(n=4)**

1. Fu ZZ. Analysis of risk factors for in-hospital adverse events in patients with acute coronary syndrome and the construction of a prediction model. Lanzhou University. 2024.
2. Baustert L, Boethig D, Görler A, Kühn C, Weymann A, Schmack B, Popov AF, Ruhparwar A, Wiegmann B. The Hannover Postinfarction Ventricular Septal Rupture Score: A New Scoring System Predicting 30-Day Mortality. CJC Open. 2025 Jan 2;7(4):456-464. doi: 10.1016/j.cjco.2024.12.013. PMID: 40433127; PMCID: PMC12105758.
3. Zhang Z, Liu Y, Cheng Q, Zhang J, Gao C. Development of a nomogram to predict 30-day mortality in patients with post-infarction ventricular septal rupture. Sci Rep. 2024 Jul 31;14(1):17690. doi: 10.1038/s41598-024-68792-y. PMID: 39085556; PMCID: PMC11292003.
4. Fu YF, Liang ZS, Feng WC. Construction of a prediction model of ultrasound indicators for mortality risk within 7 days in patients with acute myocardial infarction and ventricular septal rupture. Chin Crit Care Med. 2024,36(11):1169-1173.

**Not peer-reviewed** **(n=1)**

[1]Simsek B, Kostantinis S, Karacsonyi J, Brilakis ES. Can we predict cardiac rupture in patients with ST-segment elevation myocardial infarction? J Thorac Dis. 2022 Jul;14(7):2451-2453. doi: 10.21037/jtd-22-655. PMID: 35928604; PMCID: PMC9344428.

**Review** **(n=1)**

[1]Reardon MJ, Carr CL, Diamond A, Letsou GV, Safi HJ, Espada R, Baldwin JC. Ischemic left ventricular free wall rupture: prediction, diagnosis, and treatment. Ann Thorac Surg. 1997 Nov;64(5):1509-13. doi: 10.1016/S0003-4975(97)00776-5. PMID: 9386744.

**Irrelevant research focus** **(n=1)**

[1]Liu CY, Yang X, Chen YH. Risk Prediction Model of Cardiac Rupture Based on Weighted Bayesian Network[J].Computer & Digital Engineering,2025,53(03):684-691.
