# Supplementary material for: Risk prediction models for cardiac rupture after acute myocardial infarction: a systematic review and meta-analysis
Source: Front Cardiovasc Med. 2026 Feb 11;13:1721103. doi: 10.3389/fcvm.2026.1721103 (PMC12933645; doi:10.3389/fcvm.2026.1721103)
Supplement: Supplementary file 8 [file Table8.docx]

**Supplementary Table S8.** **Risk of bias assessment tables and key for risk of bias assessment**

**Risk of bias assessment: population, predictors and outcomes (based on PROBAST)**

| **Study** | **Model** | | **Appropriate data source?** | **In/exclusions of participants appropriate?** | **Predictors defined and assessed in similar way for all participants?** | **Predictor assessment made without knowledge of outcome data?** | **All predictors available at the time the model is intended to be used?** | **Outcome determined appropriately?** | **Pre-defined/standard outcome definition?** | **Predictors excluded from the outcome definition?** | **Outcome defined and determined in a similar way for all participants?** | **Outcome determined without knowledge of predictor information?** | **Appropriate time interval between predictor and outcome assessment?** |
| --- | --- | --- | --- | --- | --- | --- | --- | --- | --- | --- | --- | --- | --- |
| Abulimiti A *et al.* |  | **DEV √**  INT VAL X  EXT VAL X  U/M X  COMP X | NI | PY | PY | PY | Y | Y | PY | Y | PY | NI | NI |
| Bai Y *et al.* |  | **DEV √**  **INT VAL √**  EXT VAL X  U/M X  COMP X | PY | PY | PY | PY | Y | Y | PY | Y | PY | NI | NI |
| Fu Y *et al.* | **GRACE** | **DEV √**  INT VAL X  **EXT VAL √**  U/M X  **COMP √** | PY | NI | PY | PY | Y | Y | PY | Y | PY | NI | NI |
| Luo Y *et al.* |  | **DEV √**  INT VAL X  **EXT VAL √**  U/M X  COMP X | PY | PY | PY | PY | Y | Y | PY | Y | PY | NI | NI |
| Qian G *et al.* | **GRACE** | **DEV √**  **INT VAL √**  **EXT VAL √**  U/M X  **COMP √** | PY | PY | PY | PY | Y | Y | PY | Y | PY | NI | NI |
| Wu P *et al.* |  | **DEV √**  **INT VAL √**  EXT VAL X  U/M X  COMP X | PY | PY | Y | PY | Y | Y | PY | Y | PY | NI | PY |
| Wubuli D *et al.* |  | **DEV √**  **INT VAL √**  **EXT VAL √**  U/M X  COMP X | PY | NI | PY | NI | Y | Y | PY | Y | Y | NI | NI |
| Yan L *et al.* |  | **DEV √**  **INT VAL √**  **EXT VAL √**  U/M X  **COMP √** | PY | NI | Y | PY | Y | Y | PY | Y | Y | NI | PY |
| Yisimitila T *et al.* |  | **DEV √**  INT VAL X  EXT VAL X  U/M X  COMP X | NI | PY | PY | NI | Y | Y | PY | Y | PY | NI | NI |
| Zhang C *et al.* |  | **DEV √**  **INT VAL √**  EXT VAL X  U/M X  COMP X | PY | PY | PY | PY | Y | Y | PY | Y | PY | NI | NI |

AF = atrial fibrillation; CABG = coronary artery bypass graft; CAD = coronary artery disease; COMP=study which compares two or more models; DEV=model development study; EDW = Enterprise Data Warehouse; EXT VAL =study with external validation of a model; INT VAL= study with internal model validation; LVEF=left ventricular ejection fraction; N=no; NEI=not enough information; NI=no information; PCI = Percutaneous Coronary Intervention; PN=probably no; PY=probably yes; U/M =study which updates or modifies a model; Y=yes.

* relates to postoperative stroke, postoperative potassium, postoperative low cardiac output, length of hospital stay, postoperative IABP assistance, ICU stay time, ventilator support time, postoperative β-blocker treatment, postoperative ACEI treatment, postoperative potassium supplementation, postoperative NSAIDs treatment, as a variable for prediction of POAF, so variables cannot be used pre-procedurally

**Risk of bias assessment: analysis (based on PROBAST)**

| **Study** | **Model** | **Was there a reasonable number of participants with the outcome?** | **Were continuous and categorical predictors handled appropriately? For validation: was model evaluated as originally fitted?** | **Were all enrolled participants included in the analysis?** | **Were participants with missing data handled appropriately?** | **Was selection of predictors based on univariate analysis avoided? (DEV only)** | **Were complexities in the data (e.g. censoring, competing risks, sampling of control participants) accounted for appropriately?*** | **Were relevant model performance measures evaluated appropriately?** | **Were model overfitting and optimism in model performance accounted for? (DEV only)** | **Do predictors and their assigned weights in the final model correspond to the results from the reported multivariable analysis? (DEV only)** | **Where applicable: Appropriate quantification of added value (0ne score compared to another)? Appropriate method of updating model?** |
| --- | --- | --- | --- | --- | --- | --- | --- | --- | --- | --- | --- |
| Abulimiti A *et al.* | **DEV √**  INT VAL X  EXT VAL X  U/M X  COMP X | N  37 candidate variables; 37 events. 1 EPV. | PN | NI | NI | N  Variables chosen on basis of univariate analysis. | PN  Logistic regression model was used;  better to use time-to-event analysis + censoring. | Y  (NB assessment of calibration based on Hosmer-Lemeshow test which has limited power). | N  No internal validation. | Y | N/A |
| Bai Y *et al.* | **DEV √**  **INT VAL √**  EXT VAL X  U/M X  COMP X | N  20 candidate variables; 55 events. 3 EPV. | PN | NI  23/11603 (0.20%) excluded for incomplete record | N | N  Variables chosen on basis of univariate analysis. | PN  Logistic regression model was used;  better to use time-to-event analysis + censoring. | Y  (NB assessment of calibration based on Hosmer-Lemeshow test which has limited power). | Y  Hold-out validation | Y | N/A |
| Fu Y *et al.*  DEV | **GRACE**  **DEV √**  INT VAL X  **EXT VAL √**  U/M X  **COMP √** | N  41 candidate variables; 53 events. 1 EPV. | PN | NI  6/7985 (0.08%) excluded for incomplete record | N | N  Variables chosen on basis of univariate analysis. | PN  Logistic regression model was used;  better to use time-to-event analysis + censoring. | Y  (NB assessment of calibration based on Hosmer-Lemeshow test which has limited power). | N  No internal validation. | Y | N/A |
| Fu Y *et al.*  EXT VAL  (GRACE) |  | PY for EXT VAL  53 events; 7985 sample size | Y |  |  |  | N/A  No model refitting |  |  |  |  |
| Luo Y *et al.*  DEV | **DEV √**  INT VAL X  **EXT VAL √**  U/M X  COMP X | N  30 candidate variables; 86 events. 3 EPV. | PN | NI | NI | N  Variables chosen on basis of univariate analysis. | PN  Logistic regression model was used;  better to use time-to-event analysis + censoring. | Y  (NB assessment of calibration based on Hosmer-Lemeshow test which has limited power). | N  No internal validation. | Y | N/A |
| Luo Y *et al.*  EXT VAL |  | N for EXT VAL  40 events; 151 sample size | Y |  |  |  | N/A  No model refitting |  |  |  |  |
| Qian G *et al.*  DEV | **GRACE**  **DEV √**  **INT VAL √**  **EXT VAL √**  U/M X  **COMP √** | N  26 candidate variables; 158 events. 6 EPV. | PN | NI | NI | N  Variables chosen on basis of univariate analysis. | PN  Logistic regression model was used;  better to use time-to-event analysis + censoring. | N  No calibration measures reported. | Y  Temporal validation | Y | N/A |
| Qian G *et al.*  EXT VAL  (GRACE) |  | PY for INT VAL  80 events; 3779 sample size | Y |  |  |  | N/A  No model refitting |  |  |  |  |
| Wu P *et al.* | **DEV √**  **INT VAL √**  EXT VAL X  U/M X  COMP X | N  48 candidate variables; 91 events. 2 EPV. | Y | NI | NI | Y  LASSO regression analysis to identify variables | PN  Logistic regression model was used;  better to use time-to-event analysis + censoring. | Y  (NB assessment of calibration based on Hosmer-Lemeshow test which has limited power). | Y  Bootstrap | Y | N/A |
| Wubuli D *et al.* | **DEV √**  **INT VAL √**  EXT VAL X  U/M X  COMP X | N  36 candidate variables; 167 events. 5 EPV. | PN | NI | NI | N  Variables chosen on basis of univariate analysis. | PN  Logistic regression model was used;  better to use time-to-event analysis + censoring. | Y  (NB assessment of calibration based on Hosmer-Lemeshow test which has limited power). | Y  Hold-out validation | Y | N/A |
| Yan L *et al.*  DEV | **DEV √**  **INT VAL √**  **EXT VAL √**  U/M X  **COMP √** | N  21 candidate variables; 38 events. 2 EPV. | PN | NI  32/3756 (0.85%) excluded for incomplete record or pre-admission cardiac rupture. | NI | N  Variables chosen on basis of univariate analysis. | PN  Logistic regression model was used;  better to use time-to-event analysis + censoring. | Y  (NB assessment of calibration based on Hosmer-Lemeshow test which has limited power). | Y  Hold-out validation and Bootstrap | Y | N/A |
| Yan L *et al.*  EXT VAL  (Qian G) |  | N for EXT VAL  18 events; 108 sample size | Y |  |  |  | N/A  No model refitting |  |  |  |  |
| Yisimitila T *et al.* | **DEV √**  INT VAL X  EXT VAL X  U/M X  COMP X | N  22 candidate variables; 38 events. 2 EPV. | NI | NI | NI | N  Variables chosen on basis of univariate analysis. | PN  Logistic regression model was used;  better to use time-to-event analysis + censoring. | N  No calibration measures reported. | N  No internal validation. | NI | N/A |
| Zhang C *et al.*  DEV | **DEV √**  **INT VAL √**  EXT VAL X  U/M X  COMP X | N  25 candidate variables; 53 events. 2 EPV. | PN | NI | NI | N  Variables chosen on basis of univariate analysis. | PN  Logistic regression model was used;  better to use time-to-event analysis + censoring. | Y  (NB assessment of calibration based on Hosmer-Lemeshow test which has limited power). | Y  Hold-out validation | Y | N/A |

COMP=study which compares two or more models; DEV=model development study; EPV=events per variable; EXT VAL =study with external validation of a model; INT VAL= study with internal model validation; N=no; NEI=not enough information; NI=no information; PN=probably no; PY=probably yes; U/M =study which updates or modifies a model; Y=yes.

**Risk of bias assessment –key (informed by PROBAST)**

| *Appropriate data source?* | Y if prospective cohort with consecutive AMI patients (or all within a specified timeframe).  PY if retrospective analysis with consecutive patients or prospective cohort (limited details).  NI if single-center study with insufficient information on patient selection.  PNif subgroup analysis from a larger AMI cohort. |
| --- | --- |
| *Were all inclusions and exclusion appropriate?* | PY if inclusion criteria were clearly defined and appropriate for review question (e.g., studies on risk prediction models for cardiac rupture post-AMI).  NI if eligibility criteria were inconsistently reported (e.g., mixed for AMI diagnosis, model development, or validation).  PN if exclusion criteria were poorly documented (e.g., prior cardiac rupture, cardiogenic shock). |
| *Applicability* –do participants/setting match the review question? | Not included in table. Most studies focused on post infarction cardiac rupture prediction, but some models were derived from mixed ACS populations and later applied to AMI. These are flagged for "indirectness" (GRADE) in the discussion. |
| *Predictors defined and assessed in similar way for all participants?* | Y if reference made to standard criteria used in all patients.  PY if single centre. For some criteria there is a standard way of measuring (e.g. echocardiographic measures, biomarkers), others are not prone to measurement issues (e.g. age, sex). Some information is unlikely to be reported, e.g. how co-morbidities were defined.  PY if states that methods performed in accordance with AMI management.  PY if multicentre but standardised protocol.  NI if not able to tell if single centre and no other information.  N if specific statement that variables were measured in different ways. |
| *Predictor assessment made without knowledge of outcome data?* | Y if clear that all predictors measured before cardiac rupture (e.g., admission labs, imaging).  PY if appears that (at least some) predictors were measured at admission. Less important for fixed predictors (age, sex).  NI if no details on timing of predictor assessment. |
| *All predictors available at the time the model is intended to be used?* | Y if all predictors available pre-rupture (e.g., clinical variables, imaging).  N if included post-AMI events (e.g., reinfarction) unknown at baseline. |
| *Was the outcome determined appropriately?* | Y if autopsy, surgical confirmation, or rigorous imaging (e.g., echocardiography/CT).  PY if clinical diagnosis + supportive imaging (no strict protocol).  NI if medical record review without active surveillance. |
| *Standard outcome definition?* | Y if cardiac rupture confirmed within 30 days post-AMI.  PY if defined as "cardiac rupture" without duration specified.  NI if no clear definition. |
| *Were predictors excluded from the outcome definition?* | This is always Y. Predictors do not form part of outcome assessment. |
| *Was the outcome defined and determined in a similar way for all participants?* | Y if explicit statement.  PY if the standard definition given and/or single centre. |
| *Was the outcome determined without knowledge of predictor information?* | NI if no information on blinding.  N if outcome assessors aware of predictor status (e.g., high-risk biomarkers). |
| *Appropriate time interval between predictor and outcome assessment?* | Y if ≥30-day follow-up covering high-risk period.  PY if Likely adequate (median follow-up reported).  NI –no (or unclear) information given on length of follow-up.  PN if limited to in-hospital events only.  This was somewhat subjective as sometimes only a mean or median (with or without a range was stated) and the minimum follow-up period for all patients was not known. |
| *Was there a reasonable number of participants with the outcome?* | Development studies  Y -if >20 events per variable for candidate predictors  PY –if > 10 events per variable  NI- no details or unclear how many candidate variables  PN- if number of candidate variables unclear but small sample size  N -<10 events per variable  Validation studies  Y-at least 100 participants with outcome.  PY –if events not stated but very large sample size (e.g. >1000)  NI-number of events not stated  N-less than 100 participants with outcome |
| *Were continuous and categorical predictors handled appropriately?* | Development studies  Y –if no dichotomisation of continuous predictors based on study data or if cut-off predefined (widely accepted) rather than based on the data  NI- no details  PN - appears that one or more cut-offs based on study data  N-if dichotomisation of one or more continuous predictors based on study data (and no adjustment by applying internal validation and shrinkage techniques)  Validation studies  Y- if model being used as originally fitted-same dichotomisation and cut-offs. Using equation/model as created.  PY- appears that same model is being used but not explicit  PN-appears that some model variables have been changed  N-some model variables (or cut-offs) clearly changed |
| *Were all enrolled participants included in the analysis?* | Y- explicit statement that all patients were included in analysis (or if some were excluded –that the characteristics were similar to the included)  NI-no details, or small proportion (<10%) of patients excluded but no information on similarity of patient characteristics between in- and excluded  N-clear statement that >10% patients lost to follow-up (or with missing predictor information) were excluded from the analysis and/or that patient characteristics differed between in-and excluded  Note that sometimes availability of variable or outcome data was as an eligibility criterion. |
| *Were participants with missing data handled appropriately?* | 1. an explicit statement that all enrolled patients were included in the final analysis or studies reported an appropriate method for handling missing data, e.g. by using multiple imputation.   NI- no details on handling of missing data  N- patients lost to follow-up simply excluded (and no details on similarity of patients characteristics between in- and excluded)  N/A in studies where all patients had been included in analysis (or if excluded had similar characteristics to included). |
| *Was selection of predictors based on univariate analysis avoided? (DEV only)* | Y- if predictors identified through multivariate analysis  NI –no details on how predictors were selected  N- if predictors selected on the basis of univariable analysis before multivariable modelling |
| *Were complexities in the data (e.g. censoring, competing risks, sampling of control participants) accounted for appropriately?* | Development studies  Y- time to event analysis used (e.g. Cox analysis)  NI-no details on type of analysis  PN- logistic regression model used/insufficient information  N-no time-to event analysis used  Validation studies  N/A as none of the validation studies undertook calibration and model refitting. |
| *Were relevant model performance measures evaluated appropriately?* | Y-both a discrimination and calibration statistic reported  N-only one of the above or none presented |
| *Were model overfitting and optimism in model performance accounted for? (DEV only)* | Y-a form of internal validation included (e.g. bootstrapping or cross-validation); where included should adjust or shrink the model predictive performance estimates and predictor effects in the final model  PN- a split sample approach used with >20 events per candidate variable  N-no form of internal validation or a split sample approach with <20 events per candidate variable |
| *Do predictors and their assigned weights in the final model correspond to the results from the reported multivariable analysis? (DEV only)* | Y-regression co-efficients used to estimate contribution of each variable to the risk  PY- score scale based on regression co-efficients (with no further details)  NI-no information on how weights assigned  N-inappropriate method, e.g. assigning points based on relative risk or degree of separation of Kaplan-Meier curves |

DEV=model development study; N=no; NEI=not enough information; NI=no information; PN=probably no; PY=probably yes; Y=yes.
