# Supplementary material for: Risk prediction models for cardiac rupture after acute myocardial infarction: a systematic review and meta-analysis
Source: Front Cardiovasc Med. 2026 Feb 11;13:1721103. doi: 10.3389/fcvm.2026.1721103 (PMC12933645; doi:10.3389/fcvm.2026.1721103)
Supplement: Supplementary file 9 [file Table9.docx]

**Supplementary Table S8. Risk of bias appraisal results of eligible articles adapted from PROBAST**

| **Study** | **ROB** | | | | **Applicability** | | | **Overall** | |
| --- | --- | --- | --- | --- | --- | --- | --- | --- | --- |
|  | **Participants** | **Predictors** | **Outcome** | **Analysis** | **Participants** | **Predictors** | **Outcome** | **ROB** | **Applicability** |
| Abulimiti A et al. 2022 | ? | ＋ | ? | － | ＋ | ＋ | ＋ | － | ＋ |
| Bai Y et al. 2024 | ＋ | ＋ | ? | － | ＋ | ＋ | ＋ | － | ＋ |
| Fu Y et al. 2019 | ? | ＋ | ? | － | ＋ | ＋ | ＋ | － | ＋ |
| Luo Y et al. 2022 | ＋ | ＋ | ? | － | ＋ | ＋ | ＋ | － | ＋ |
| Qian G et al. 2017 | ＋ | ＋ | ? | － | ＋ | ＋ | ＋ | － | ＋ |
| Wu P et al. 2024 | ＋ | ＋ | ? | － | ＋ | ＋ | ＋ | － | ＋ |
| Wubuli D et al. 2020 | ? | ? | ? | － | ＋ | ＋ | ＋ | － | ＋ |
| Yan L et al. 2021 | ? | ＋ | ? | － | ＋ | ＋ | ＋ | － | ＋ |
| Yisimitila T et al 2023 | ? | ? | ? | － | ＋ | ＋ | ＋ | － | ＋ |
| Zhang C et al. 2024 | ＋ | ＋ | ? | － | ＋ | ＋ | ＋ | － | ＋ |

“+” indicates low ROB/low concern regarding applicability; “－” indicates high ROB/low concern regarding applicability; and “?” indicates unclear ROB/low concern regarding applicability

ROB risk of bias
