# Supplementary material for: Risk prediction models for cardiac rupture after acute myocardial infarction: a systematic review and meta-analysis
Source: Front Cardiovasc Med. 2026 Feb 11;13:1721103. doi: 10.3389/fcvm.2026.1721103 (PMC12933645; doi:10.3389/fcvm.2026.1721103)
Supplement: Supplementary file 10 [file Image1.pdf]

A

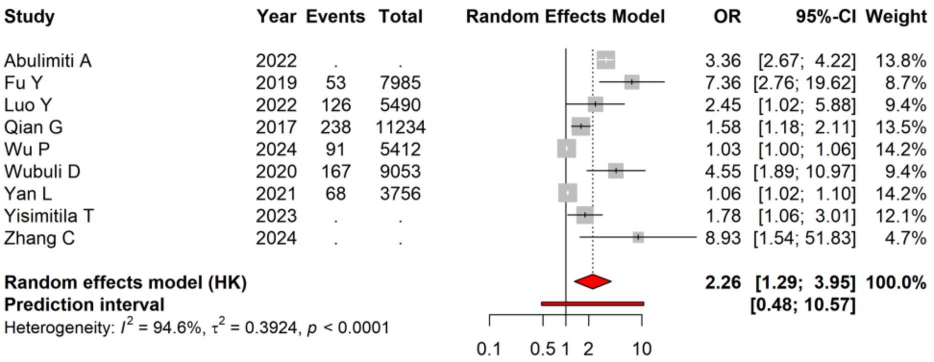
Study Year Events Total Random Effects Model OR 95%-CI Weight
Abulimiti A 2022 . . 3.36 [2.67; 4.22] 13.8%
Fu Y 2019 53 7985 7.36 [2.76; 19.62] 8.7%
Luo Y 2022 126 5490 2.45 [1.02; 5.88] 9.4%
Qian G 2017 238 11234 1.58 [1.18; 2.11] 13.5%
Wu P 2024 91 5412 1.03 [1.00; 1.06] 14.2%
Wubuli D 2020 167 9053 4.55 [1.89; 10.97] 9.4%
Yan L 2021 68 3756 1.06 [1.02; 1.10] 14.2%
Yisimitila T 2023 . . 1.78 [1.06; 3.01] 12.1%
Zhang C 2024 . . 8.93 [1.54; 51.83] 4.7%
Random effects model (HK) 2.26 [1.29; 3.95] 100.0%
Prediction interval [0.48; 10.57]
Heterogeneity: I^2 = 94.6%, τ^2 = 0.3924, p < 0.0001
0.1 0.5 1 2 10

B

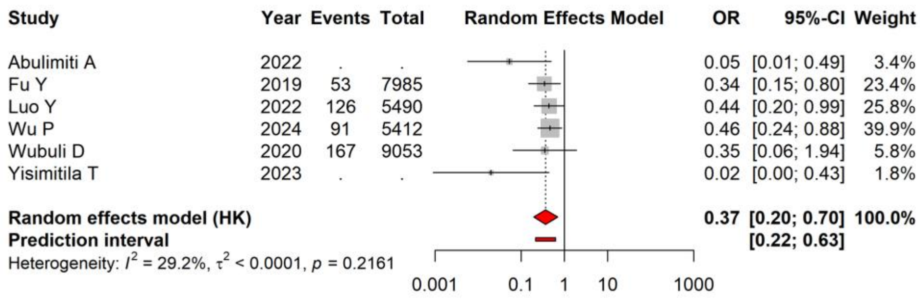
Study Year Events Total Random Effects Model OR 95%-CI Weight
Abulimiti A 2022 . . 0.05 [0.01; 0.49] 3.4%
Fu Y 2019 53 7985 0.34 [0.15; 0.80] 23.4%
Luo Y 2022 126 5490 0.44 [0.20; 0.99] 25.8%
Wu P 2024 91 5412 0.46 [0.24; 0.88] 39.9%
Wubuli D 2020 167 9053 0.35 [0.06; 1.94] 5.8%
Yisimitila T 2023 . . 0.02 [0.00; 0.43] 1.8%
Random effects model (HK) 0.37 [0.20; 0.70] 100.0%
Prediction interval [0.22; 0.63]
Heterogeneity: I^2 = 29.2%, τ^2 < 0.0001, p = 0.2161
0.001 0.1 1 10 1000

C

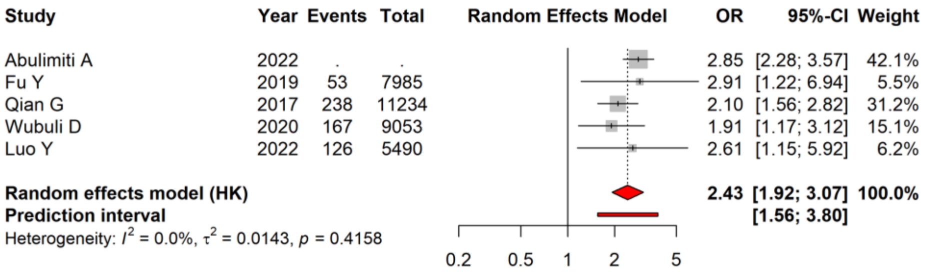
Study Year Events Total Random Effects Model OR 95%-CI Weight
Abulimiti A 2022 . . 2.85 [2.28; 3.57] 42.1%
Fu Y 2019 53 7985 2.91 [1.22; 6.94] 5.5%
Qian G 2017 238 11234 2.10 [1.56; 2.82] 31.2%
Wubuli D 2020 167 9053 1.91 [1.17; 3.12] 15.1%
Luo Y 2022 126 5490 2.61 [1.15; 5.92] 6.2%
Random effects model (HK) 2.43 [1.92; 3.07] 100.0%
Prediction interval [1.56; 3.80]
Heterogeneity: I^2 = 0.0%, τ^2 = 0.0143, p = 0.4158
0.2 0.5 1 2 5

D

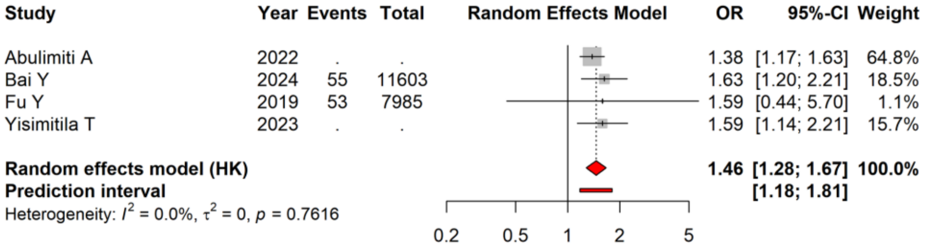
Study Year Events Total Random Effects Model OR 95%-CI Weight
Abulimiti A 2022 . . 1.38 [1.17; 1.63] 64.8%
Bai Y 2024 55 11603 1.63 [1.20; 2.21] 18.5%
Fu Y 2019 53 7985 1.59 [0.44; 5.70] 1.1%
Yisimitila T 2023 . . 1.59 [1.14; 2.21] 15.7%
Random effects model (HK) 1.46 [1.28; 1.67] 100.0%
Prediction interval [1.18; 1.81]
Heterogeneity: I^2 = 0.0%, τ^2 = 0, p = 0.7616
0.2 0.5 1 2 5

E

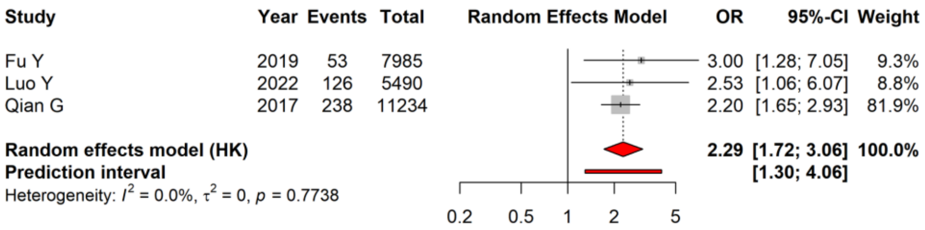
Study Year Events Total Random Effects Model OR 95%-CI Weight
Fu Y 2019 53 7985 3.00 [1.28; 7.05] 9.3%
Luo Y 2022 126 5490 2.53 [1.06; 6.07] 8.8%
Qian G 2017 238 11234 2.20 [1.65; 2.93] 81.9%
Random effects model (HK) 2.29 [1.72; 3.06] 100.0%
Prediction interval [1.30; 4.06]
Heterogeneity: I^2 = 0.0%, τ^2 = 0, p = 0.7738
0.2 0.5 1 2 5

F

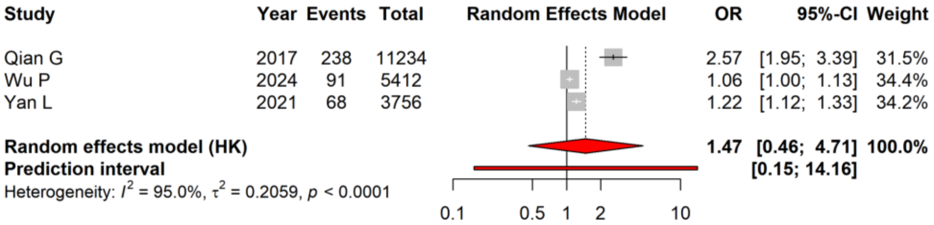
Study Year Events Total Random Effects Model OR 95%-CI Weight
Qian G 2017 238 11234 2.57 [1.95; 3.39] 31.5%
Wu P 2024 91 5412 1.06 [1.00; 1.13] 34.4%
Yan L 2021 68 3756 1.22 [1.12; 1.33] 34.2%
Random effects model (HK) 1.47 [0.46; 4.71] 100.0%
Prediction interval [0.15; 14.16]
Heterogeneity: I^2 = 95.0%, τ^2 = 0.2059, p < 0.0001
0.1 0.5 1 2 10

G

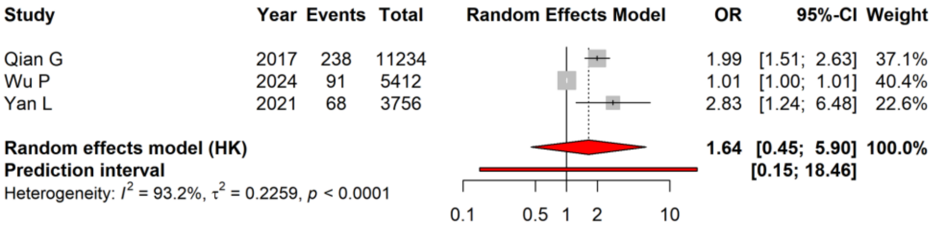
Study Year Events Total Random Effects Model OR 95%-CI Weight
Qian G 2017 238 11234 1.99 [1.51; 2.63] 37.1%
Wu P 2024 91 5412 1.01 [1.00; 1.01] 40.4%
Yan L 2021 68 3756 2.83 [1.24; 6.48] 22.6%
Random effects model (HK) 1.64 [0.45; 5.90] 100.0%
Prediction interval [0.15; 18.46]
Heterogeneity: I^2 = 93.2%, τ^2 = 0.2259, p < 0.0001
0.1 0.5 1 2 10

H

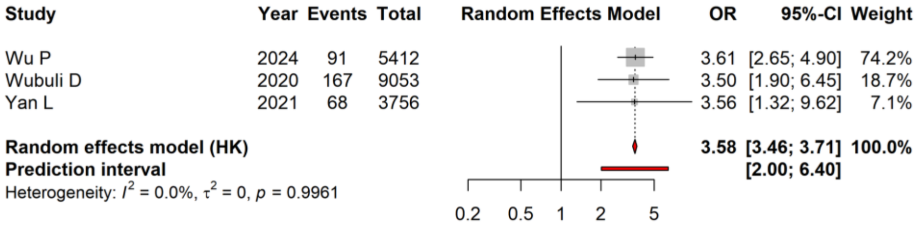
Study Year Events Total Random Effects Model OR 95%-CI Weight
Wu P 2024 91 5412 3.61 [2.65; 4.90] 74.2%
Wubuli D 2020 167 9053 3.50 [1.90; 6.45] 18.7%
Yan L 2021 68 3756 3.56 [1.32; 9.62] 7.1%
Random effects model (HK) 3.58 [3.46; 3.71] 100.0%
Prediction interval [2.00; 6.40]
Heterogeneity: I^2 = 0.0%, τ^2 = 0, p = 0.9961
0.2 0.5 1 2 5

I

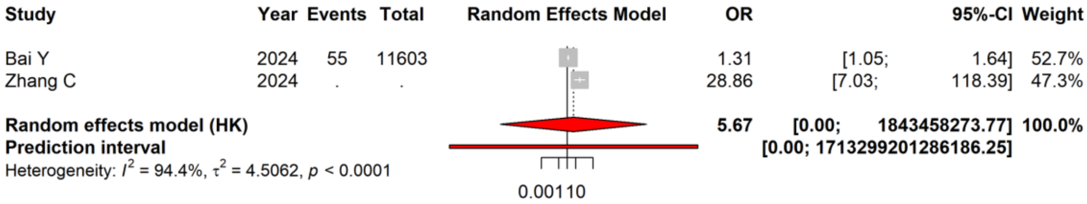
Study Year Events Total Random Effects Model OR 95%-CI Weight
Bai Y 2024 55 11603 1.31 [1.05; 1.64] 52.7%
Zhang C 2024 . . 28.86 [7.03; 118.39] 47.3%
Random effects model (HK) 5.67 [0.00; 1843458273.77] 100.0%
Prediction interval [0.00; 1713299201286186.25]
Heterogeneity: I^2 = 94.4%, τ^2 = 4.5062, p < 0.0001
0.00110

J

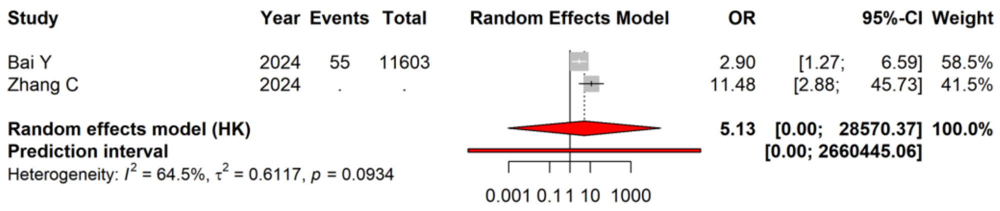

Supplementary Figures S1. Forest plot of the meta-analysis on risk factors for cardiac rupture.

(A) Forest plot for age. (B) Forest plot for emergency PCI. (C) Forest plot for female gender. (D) Forest plot for LVEF. (E) Forest plot for heart rate. (F) Forest plot for WBC.(G) Forest plot for time-to-hospital admission. (H) Forest plot for Killip grade. (I) Forest plot for CRP. (J) Forest plot for pericardial effusion.
