# Supplementary material for: Risk prediction models for cardiac rupture after acute myocardial infarction: a systematic review and meta-analysis
Source: Front Cardiovasc Med. 2026 Feb 11;13:1721103. doi: 10.3389/fcvm.2026.1721103 (PMC12933645; doi:10.3389/fcvm.2026.1721103)
Supplement: Supplementary file 11 [file Image2.pdf]

A

Study or Subgroup	log[Odds Ratio]	SE	Weight	Odds Ratio IV, Random, 95% CI
Abulimiti A 2022	-0.0726	0.0334	12.5%	0.93 [0.87, 0.99]
Bai Y 2024	-0.1532	0.0415	11.8%	0.86 [0.79, 0.93]
Fu Y 2019	-0.1708	0.039	12.0%	0.84 [0.78, 0.91]
Luo Y 2022	-0.2421	0.0614	9.9%	0.78 [0.70, 0.89]
Qian G 2017	-0.2485	0.0338	12.5%	0.78 [0.73, 0.83]
Wubuli D 2020	-0.2771	0.0539	10.7%	0.76 [0.68, 0.84]
Wu P 2024	-0.0545	0.0109	13.9%	0.95 [0.93, 0.97]
Yan L 2021	-0.1875	0.0726	8.9%	0.83 [0.72, 0.96]
Zhang C 2024	-0.3216	0.0856	7.8%	0.72 [0.61, 0.86]
**Total (95% CI)**			**100.0%**	**0.83 [0.78, 0.89]**

Heterogeneity: Tau² = 0.01; Chi² = 66.40, df = 8 (P < 0.00001); I² = 88%
Test for overall effect: Z = 5.08 (P < 0.00001)

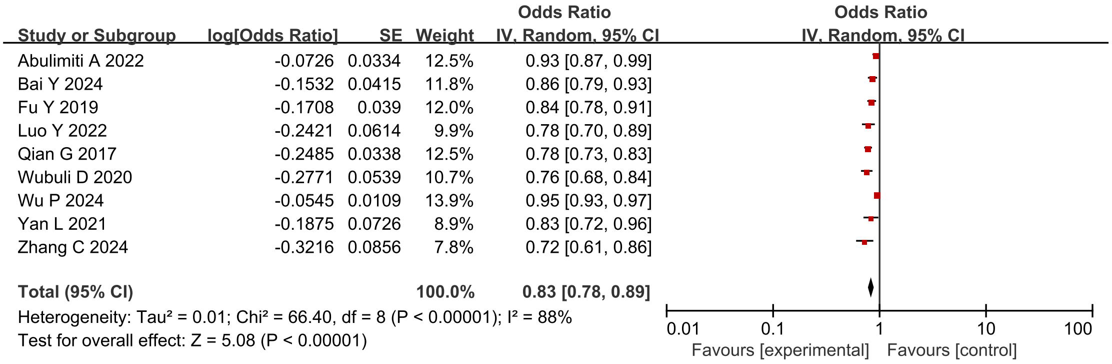

B

Study or Subgroup	log[Odds Ratio]	SE	Weight	Odds Ratio IV, Fixed, 95% CI
Abulimiti A 2022	-0.0726	0.0334	0.0%	0.93 [0.87, 0.99]
Bai Y 2024	-0.1532	0.0415	0.0%	0.86 [0.79, 0.93]
Fu Y 2019	-0.1708	0.039	31.8%	0.84 [0.78, 0.91]
Luo Y 2022	-0.2421	0.0614	0.0%	0.78 [0.70, 0.89]
Qian G 2017	-0.2485	0.0338	42.4%	0.78 [0.73, 0.83]
Wubuli D 2020	-0.2771	0.0539	16.7%	0.76 [0.68, 0.84]
Wu P 2024	-0.0545	0.0109	0.0%	0.95 [0.93, 0.97]
Yan L 2021	-0.1875	0.0726	9.2%	0.83 [0.72, 0.96]
Zhang C 2024	-0.3216	0.0856	0.0%	0.72 [0.61, 0.86]
**Total (95% CI)**			**100.0%**	**0.80 [0.77, 0.84]**

Heterogeneity: Chi² = 3.61, df = 3 (P = 0.31); I² = 17%
Test for overall effect: Z = 10.14 (P < 0.00001)

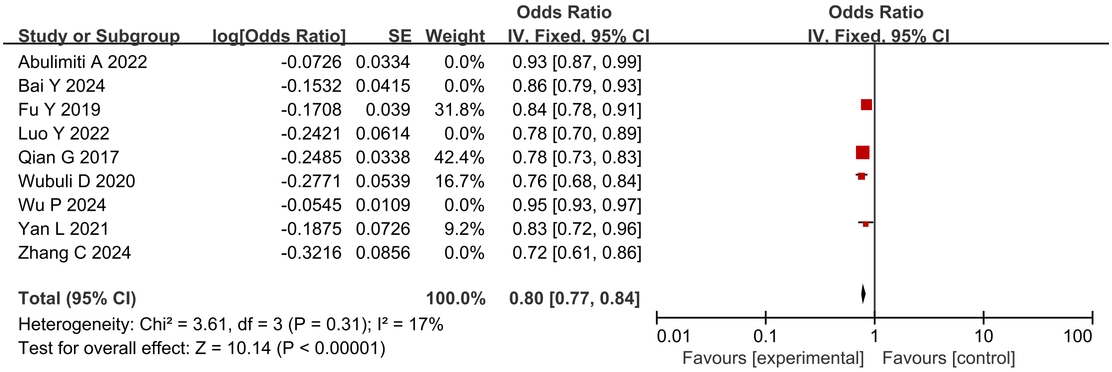

C

Study or Subgroup	log[Odds Ratio]	SE	Weight	Odds Ratio IV, Random, 95% CI
Abulimiti A 2022	-0.0726	0.0334	23.1%	0.93 [0.87, 0.99]
Bai Y 2024	-0.1532	0.0415	21.2%	0.86 [0.79, 0.93]
Fu Y 2019	-0.1708	0.039	0.0%	0.84 [0.78, 0.91]
Luo Y 2022	-0.2421	0.0614	16.6%	0.78 [0.70, 0.89]
Qian G 2017	-0.2485	0.0338	0.0%	0.78 [0.73, 0.83]
Wubuli D 2020	-0.2771	0.0539	0.0%	0.76 [0.68, 0.84]
Wu P 2024	-0.0545	0.0109	27.1%	0.95 [0.93, 0.97]
Yan L 2021	-0.1875	0.0726	0.0%	0.83 [0.72, 0.96]
Zhang C 2024	-0.3216	0.0856	12.0%	0.72 [0.61, 0.86]
**Total (95% CI)**			**100.0%**	**0.87 [0.80, 0.94]**

Heterogeneity: Tau² = 0.01; Chi² = 22.54, df = 4 (P = 0.0002); I² = 82%
Test for overall effect: Z = 3.62 (P = 0.0003)

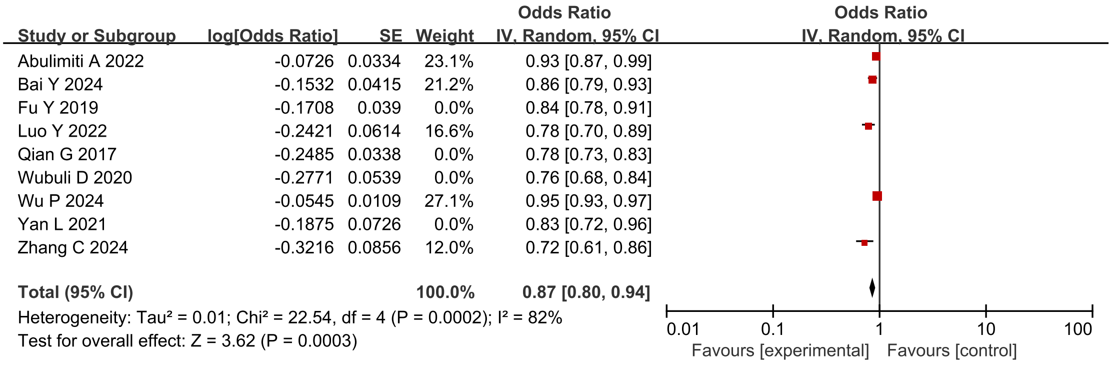

D

Study or Subgroup	log[Odds Ratio]	SE	Weight	Odds Ratio IV, Random, 95% CI
Abulimiti A 2022	-0.0726	0.0334	14.6%	0.93 [0.87, 0.99]
Bai Y 2024	-0.1532	0.0415	13.6%	0.86 [0.79, 0.93]
Fu Y 2019	-0.1708	0.039	13.9%	0.84 [0.78, 0.91]
Luo Y 2022	-0.2421	0.0614	11.1%	0.78 [0.70, 0.89]
Qian G 2017	-0.2485	0.0338	0.0%	0.78 [0.73, 0.83]
Wubuli D 2020	-0.2771	0.0539	12.0%	0.76 [0.68, 0.84]
Wu P 2024	-0.0545	0.0109	16.6%	0.95 [0.93, 0.97]
Yan L 2021	-0.1875	0.0726	9.8%	0.83 [0.72, 0.96]
Zhang C 2024	-0.3216	0.0856	8.4%	0.72 [0.61, 0.86]
**Total (95% CI)**			**100.0%**	**0.84 [0.79, 0.90]**

Heterogeneity: $Tau^2 = 0.01$; $Chi^2 = 44.38$, df = 7 ($P < 0.00001$); $I^2 = 84\%$
Test for overall effect: Z = 4.86 ($P < 0.00001$)

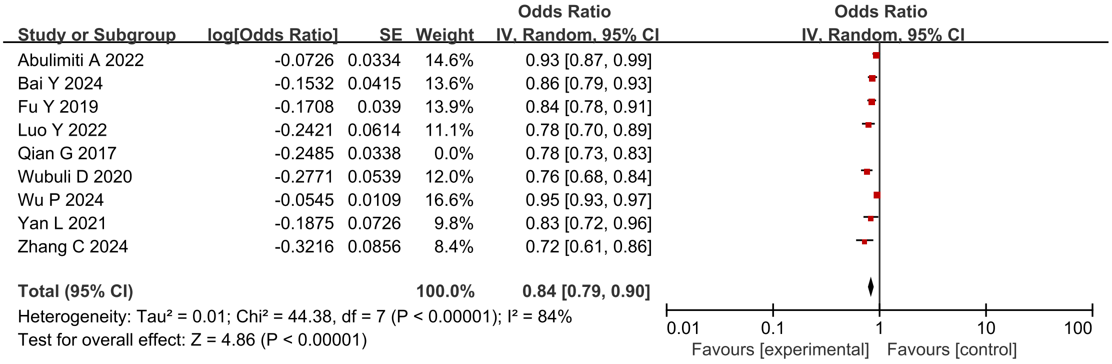

E

Study or Subgroup	log[Odds Ratio]	SE	Weight	Odds Ratio IV, Fixed, 95% CI
Abulimiti A 2022	-0.0726	0.0334	0.0%	0.93 [0.87, 0.99]
Bai Y 2024	-0.1532	0.0415	35.4%	0.86 [0.79, 0.93]
Fu Y 2019	-0.1708	0.039	40.1%	0.84 [0.78, 0.91]
Luo Y 2022	-0.2421	0.0614	16.2%	0.78 [0.70, 0.89]
Qian G 2017	-0.2485	0.0338	0.0%	0.78 [0.73, 0.83]
Wubuli D 2020	-0.2771	0.0539	0.0%	0.76 [0.68, 0.84]
Wu P 2024	-0.0545	0.0109	0.0%	0.95 [0.93, 0.97]
Yan L 2021	-0.1875	0.0726	0.0%	0.83 [0.72, 0.96]
Zhang C 2024	-0.3216	0.0856	8.3%	0.72 [0.61, 0.86]
**Total (95% CI)**			**100.0%**	**0.83 [0.79, 0.87]**

Heterogeneity: $Chi^2 = 4.11$, df = 3 ($P = 0.25$); $I^2 = 27\%$
Test for overall effect: Z = 7.64 ($P < 0.00001$)

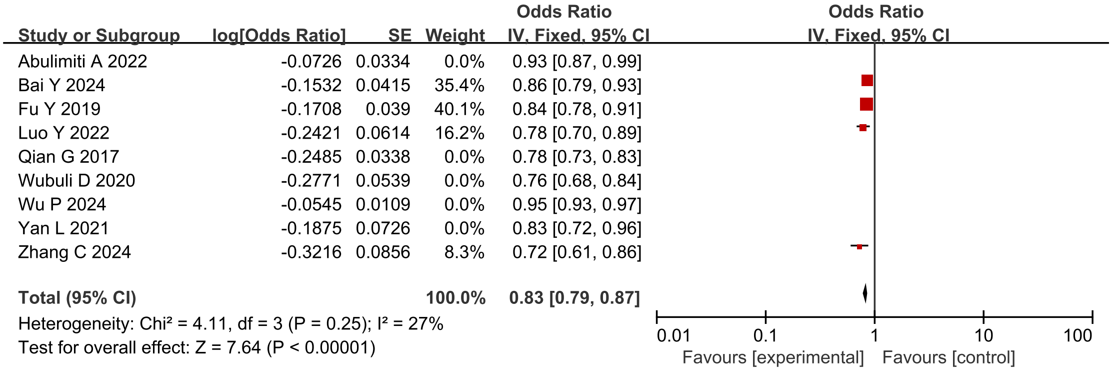

F

Study or Subgroup	log[Odds Ratio]	SE	Weight	Odds Ratio IV, Random, 95% CI
Abulimiti A 2022	-0.0726	0.0334	21.3%	0.93 [0.87, 0.99]
Bai Y 2024	-0.1532	0.0415	0.0%	0.86 [0.79, 0.93]
Fu Y 2019	-0.1708	0.039	0.0%	0.84 [0.78, 0.91]
Luo Y 2022	-0.2421	0.0614	0.0%	0.78 [0.70, 0.89]
Qian G 2017	-0.2485	0.0338	21.2%	0.78 [0.73, 0.83]
Wubuli D 2020	-0.2771	0.0539	18.5%	0.76 [0.68, 0.84]
Wu P 2024	-0.0545	0.0109	23.3%	0.95 [0.93, 0.97]
Yan L 2021	-0.1875	0.0726	15.7%	0.83 [0.72, 0.96]
Zhang C 2024	-0.3216	0.0856	0.0%	0.72 [0.61, 0.86]
**Total (95% CI)**			**100.0%**	**0.85 [0.77, 0.94]**

Heterogeneity: $Tau^2 = 0.01$; $Chi^2 = 45.92$, df = 4 ($P < 0.00001$); $I^2 = 91\%$
Test for overall effect: Z = 3.24 ($P = 0.001$)

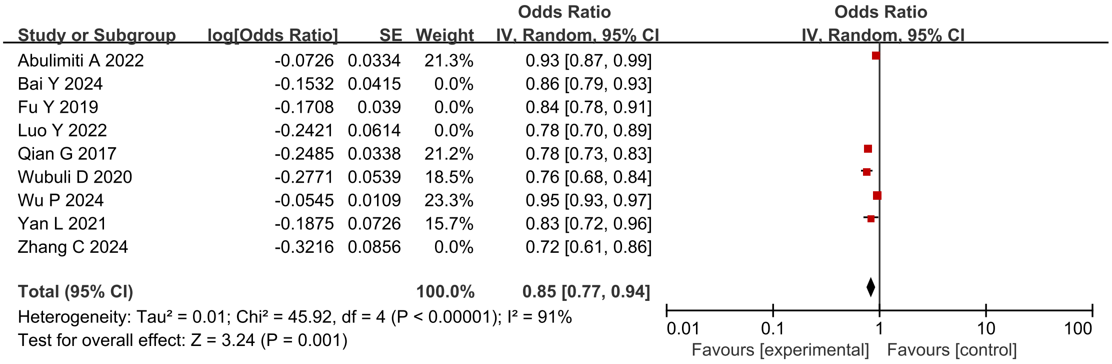

G

Study or Subgroup	log[Odds Ratio]	SE	Weight	Odds Ratio IV, Random, 95% CI
Abulimiti A 2022	-0.0726	0.0334	0.0%	0.93 [0.87, 0.99]
Bai Y 2024	-0.1532	0.0415	14.8%	0.86 [0.79, 0.93]
Fu Y 2019	-0.1708	0.039	15.0%	0.84 [0.78, 0.91]
Luo Y 2022	-0.2421	0.0614	12.7%	0.78 [0.70, 0.89]
Qian G 2017	-0.2485	0.0338	15.5%	0.78 [0.73, 0.83]
Wubuli D 2020	-0.2771	0.0539	13.5%	0.76 [0.68, 0.84]
Wu P 2024	-0.0545	0.0109	17.0%	0.95 [0.93, 0.97]
Yan L 2021	-0.1875	0.0726	11.5%	0.83 [0.72, 0.96]
Zhang C 2024	-0.3216	0.0856	0.0%	0.72 [0.61, 0.86]
**Total (95% CI)**			**100.0%**	**0.83 [0.76, 0.90]**

Heterogeneity: $Tau^2 = 0.01$; $Chi^2 = 58.95$, df = 6 ($P < 0.00001$); $I^2 = 90\%$
Test for overall effect: Z = 4.33 ($P < 0.0001$)

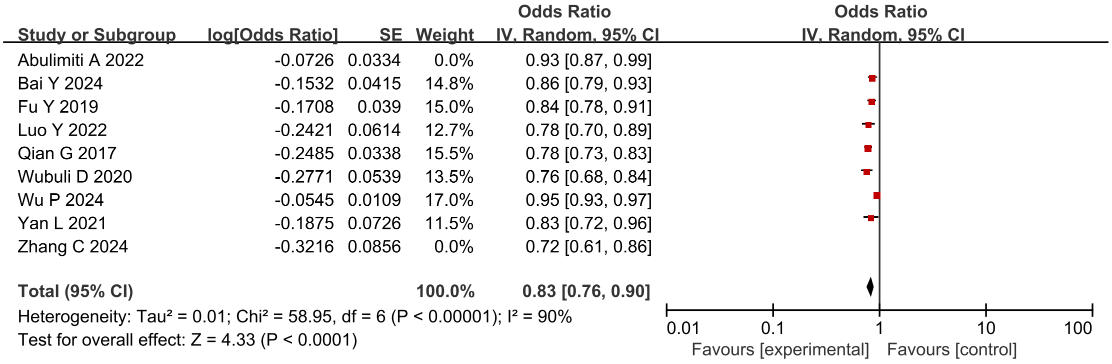

H

Study or Subgroup	log[Odds Ratio]	SE	Weight	Odds Ratio IV, Random, 95% CI
Abulimiti A 2022	-0.0726	0.0334	55.0%	0.93 [0.87, 0.99]
Bai Y 2024	-0.1532	0.0415	0.0%	0.86 [0.79, 0.93]
Fu Y 2019	-0.1708	0.039	0.0%	0.84 [0.78, 0.91]
Luo Y 2022	-0.2421	0.0614	0.0%	0.78 [0.70, 0.89]
Qian G 2017	-0.2485	0.0338	0.0%	0.78 [0.73, 0.83]
Wubuli D 2020	-0.2771	0.0539	0.0%	0.76 [0.68, 0.84]
Wu P 2024	-0.0545	0.0109	0.0%	0.95 [0.93, 0.97]
Yan L 2021	-0.1875	0.0726	0.0%	0.83 [0.72, 0.96]
Zhang C 2024	-0.3216	0.0856	45.0%	0.72 [0.61, 0.86]
**Total (95% CI)**			**100.0%**	**0.83 [0.65, 1.06]**

Heterogeneity: Tau² = 0.03; Chi² = 7.34, df = 1 (P = 0.007); I² = 86%
Test for overall effect: Z = 1.49 (P = 0.14)

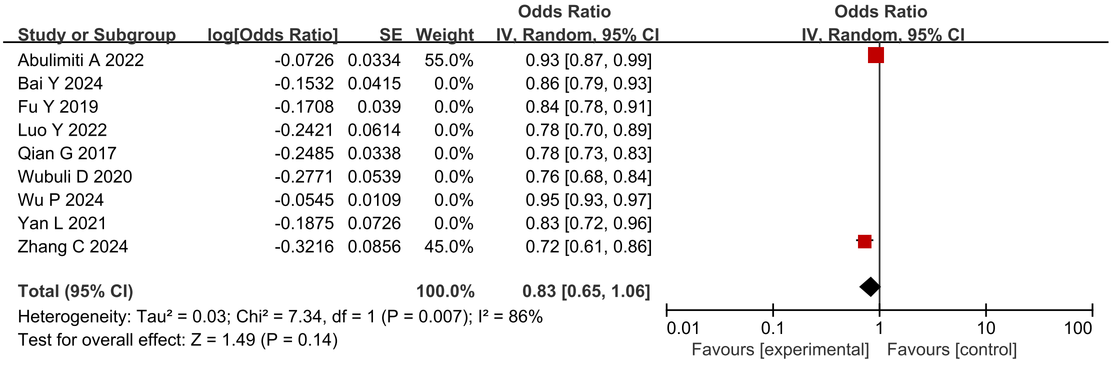

I

Study or Subgroup	log[Odds Ratio]	SE	Weight	Odds Ratio IV, Random, 95% CI
Abulimiti A 2022	-0.0726	0.0334	15.5%	0.93 [0.87, 0.99]
Bai Y 2024	-0.1532	0.0415	0.0%	0.86 [0.79, 0.93]
Fu Y 2019	-0.1708	0.039	15.0%	0.84 [0.78, 0.91]
Luo Y 2022	-0.2421	0.0614	12.4%	0.78 [0.70, 0.89]
Qian G 2017	-0.2485	0.0338	15.5%	0.78 [0.73, 0.83]
Wubuli D 2020	-0.2771	0.0539	13.3%	0.76 [0.68, 0.84]
Wu P 2024	-0.0545	0.0109	17.1%	0.95 [0.93, 0.97]
Yan L 2021	-0.1875	0.0726	11.2%	0.83 [0.72, 0.96]
Zhang C 2024	-0.3216	0.0856	0.0%	0.72 [0.61, 0.86]
**Total (95% CI)**			**100.0%**	**0.84 [0.78, 0.91]**

Heterogeneity: Tau² = 0.01; Chi² = 57.15, df = 6 (P < 0.00001); I² = 90%
Test for overall effect: Z = 4.24 (P < 0.0001)

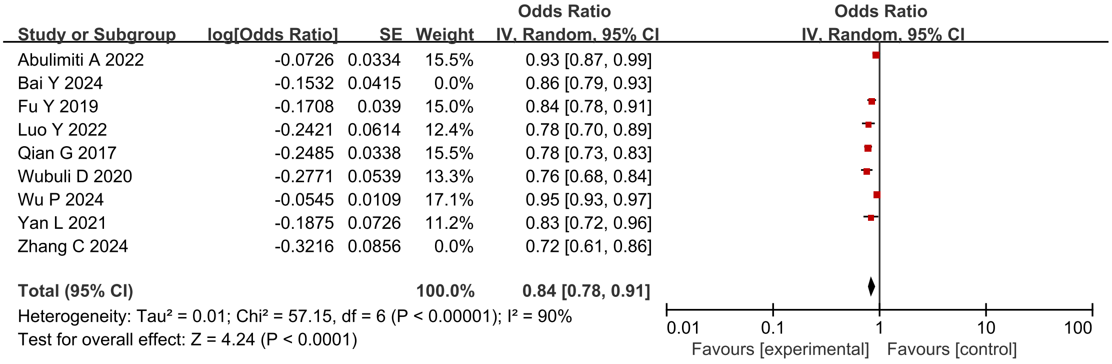

J

Study or Subgroup	log[Odds Ratio]	SE	Weight	Odds Ratio IV, Random, 95% CI
Abulimiti A 2022	-0.0726	0.0334	0.0%	0.93 [0.87, 0.99]
Bai Y 2024	-0.1532	0.0415	59.9%	0.86 [0.79, 0.93]
Fu Y 2019	-0.1708	0.039	0.0%	0.84 [0.78, 0.91]
Luo Y 2022	-0.2421	0.0614	0.0%	0.78 [0.70, 0.89]
Qian G 2017	-0.2485	0.0338	0.0%	0.78 [0.73, 0.83]
Wubuli D 2020	-0.2771	0.0539	0.0%	0.76 [0.68, 0.84]
Wu P 2024	-0.0545	0.0109	0.0%	0.95 [0.93, 0.97]
Yan L 2021	-0.1875	0.0726	0.0%	0.83 [0.72, 0.96]
Zhang C 2024	-0.3216	0.0856	40.1%	0.72 [0.61, 0.86]
**Total (95% CI)**			**100.0%**	**0.80 [0.68, 0.94]**

Heterogeneity: Tau² = 0.01; Chi² = 3.13, df = 1 (P = 0.08); I² = 68%
Test for overall effect: Z = 2.67 (P = 0.007)

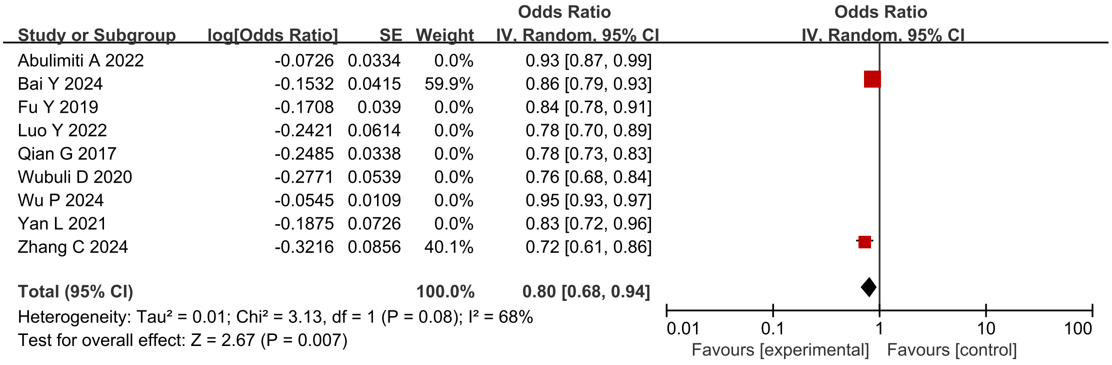

K

Study or Subgroup	log[Odds Ratio]	SE	Weight	Odds Ratio IV, Random, 95% CI
Abulimiti A 2022	-0.0726	0.0334	38.0%	0.93 [0.87, 0.99]
Bai Y 2024	-0.1532	0.0415	0.0%	0.86 [0.79, 0.93]
Fu Y 2019	-0.1708	0.039	35.6%	0.84 [0.78, 0.91]
Luo Y 2022	-0.2421	0.0614	26.4%	0.78 [0.70, 0.89]
Qian G 2017	-0.2485	0.0338	0.0%	0.78 [0.73, 0.83]
Wubuli D 2020	-0.2771	0.0539	0.0%	0.76 [0.68, 0.84]
Wu P 2024	-0.0545	0.0109	0.0%	0.95 [0.93, 0.97]
Yan L 2021	-0.1875	0.0726	0.0%	0.83 [0.72, 0.96]
Zhang C 2024	-0.3216	0.0856	0.0%	0.72 [0.61, 0.86]
**Total (95% CI)**			**100.0%**	**0.86 [0.78, 0.94]**

Heterogeneity: Tau² = 0.00; Chi² = 7.37, df = 2 (P = 0.03); I² = 73%
Test for overall effect: Z = 3.19 (P = 0.001)

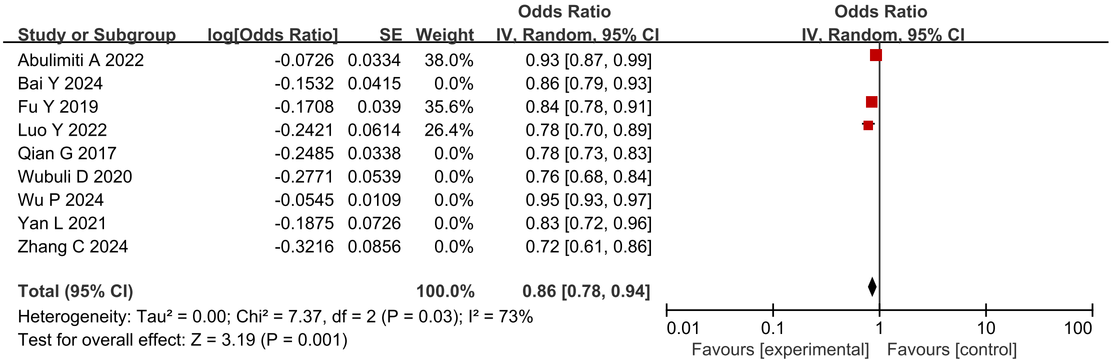

L

Study or Subgroup	log[Odds Ratio]	SE	Weight	Odds Ratio IV, Random, 95% CI
Abulimiti A 2022	-0.0726	0.0334	0.0%	0.93 [0.87, 0.99]
Bai Y 2024	-0.1532	0.0415	17.8%	0.86 [0.79, 0.93]
Fu Y 2019	-0.1708	0.039	0.0%	0.84 [0.78, 0.91]
Luo Y 2022	-0.2421	0.0614	0.0%	0.78 [0.70, 0.89]
Qian G 2017	-0.2485	0.0338	18.4%	0.78 [0.73, 0.83]
Wubuli D 2020	-0.2771	0.0539	16.5%	0.76 [0.68, 0.84]
Wu P 2024	-0.0545	0.0109	19.8%	0.95 [0.93, 0.97]
Yan L 2021	-0.1875	0.0726	14.5%	0.83 [0.72, 0.96]
Zhang C 2024	-0.3216	0.0856	13.0%	0.72 [0.61, 0.86]
**Total (95% CI)**			**100.0%**	**0.82 [0.74, 0.91]**

Heterogeneity: Tau² = 0.01; Chi² = 56.08, df = 5 (P < 0.00001); I² = 91%
Test for overall effect: Z = 3.78 (P = 0.0002)

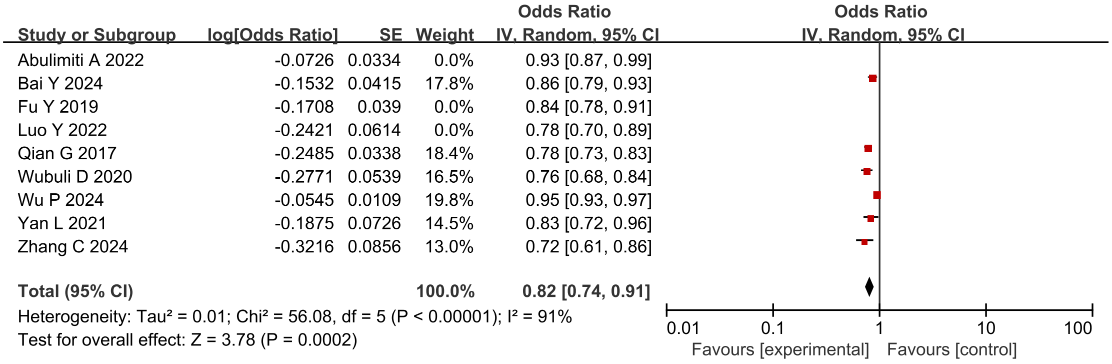

M

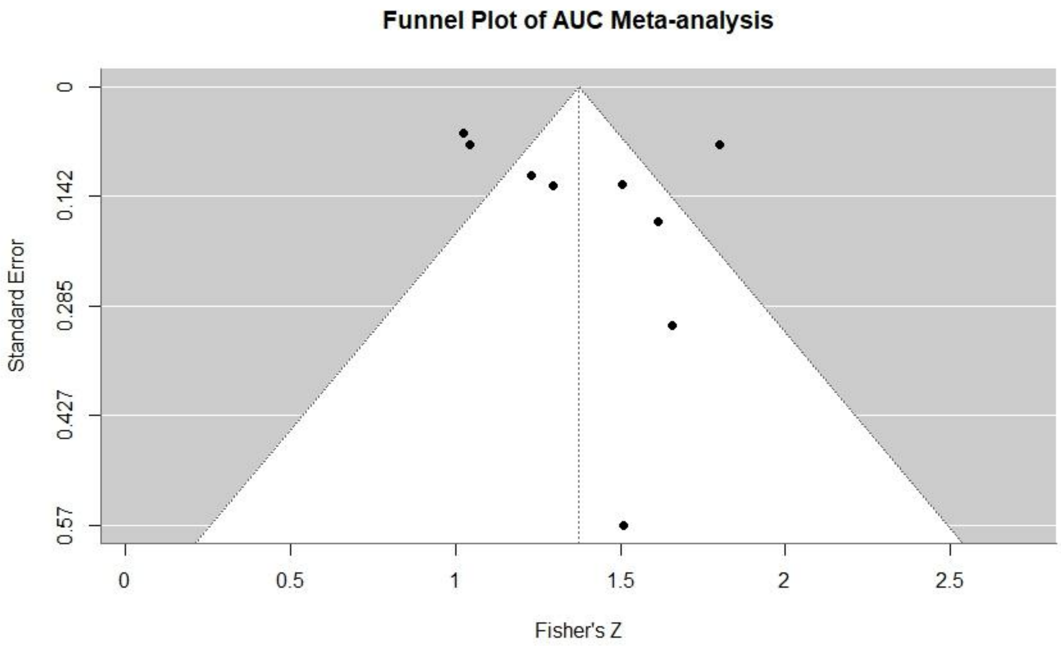

Supplementary Figures S2. Forest plot in the discrimination of models.

(A)Forest plot for all studies. (B) Forest plot for the publication data (2017-2021). (C) Forest plot for the publication data (2022-2024). (D) Forest plot for the type of study (retrospective study). (E) Forest plot for the participants (AMI). (F) Forest plot for the participants (STEMI). (G) Forest plot for the sample size (≥1000). (H) Forest plot for the sample size (<1000). (I) Forest plot for the outcome (CR). (J) Forest plot for the outcome (FWR). (K) Forest plot for no internal validation. (L) Forest plot for internal validation. (M) Funnel plot of AUC meta-analysis.
